# Current perspectives and trend of computer-aided drug design: a review and bibliometric analysis

**DOI:** 10.1097/JS9.0000000000001289

**Published:** 2024-03-19

**Authors:** Zhenhui Wu, Shupeng Chen, Yihao Wang, Fangyang Li, Huanhua Xu, Maoxing Li, Yingjian Zeng, Zhenfeng Wu, Yue Gao

**Affiliations:** a School of Pharmacy, Jiangxi University of Chinese Medicine; b School of Clinical Medicine, Jiangxi University of Chinese Medicine, Nanchang; c Beijing Institute of Radiation Medicine, Academy of Military Sciences, Beijing, People’s Republic of China

**Keywords:** artificial intelligence, bibliometrics, computer-aided drug design, COVID-19, drug discovery

## Abstract

**Aim:**

Computer-aided drug design (CADD) is a drug design technique for computing ligand–receptor interactions and is involved in various stages of drug development. To better grasp the frontiers and hotspots of CADD, we conducted a review analysis through bibliometrics.

**Methods:**

A systematic review of studies published between 2000 and 20 July 2023 was conducted following the PRISMA guidelines. Literature on CADD was selected from the Web of Science Core Collection. General information, publications, output trends, countries/regions, institutions, journals, keywords, and influential authors were visually analyzed using software such as Excel, VOSviewer, RStudio, and CiteSpace.

**Results:**

A total of 2031 publications were included. These publications primarily originated from 99 countries or regions led by the U.S. and China. Among the contributors, MacKerell AD had the highest number of articles and the greatest influence. The *Journal of Medicinal Chemistry* was the most cited journal, whereas the *Journal of Chemical Information and Modeling* had the highest number of publications.

**Conclusions:**

Influential authors in the field were identified. Current research shows active collaboration between countries, institutions, and companies. CADD technologies such as homology modeling, pharmacophore modeling, quantitative conformational relationships, molecular docking, molecular dynamics simulation, binding free energy prediction, and high-throughput virtual screening can effectively improve the efficiency of new drug discovery. Artificial intelligence-assisted drug design and screening based on CADD represent key topics that will influence future development. Furthermore, this paper will be helpful in better understanding the frontiers and hotspots of CADD.

## Graphical abstract

**Figure:**
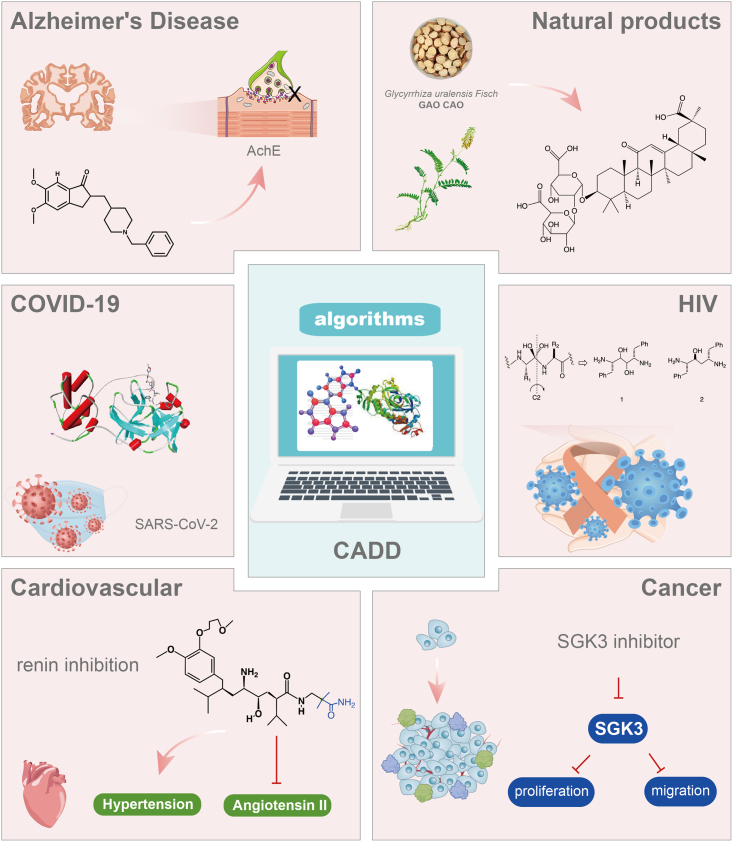

## Introduction

Highlights

- This review is the first bibliometric analysis of computer-aided drug design (CADD) in medical pharmacology.
- There has been a significant increase in the number of publications of CADD during the past 20 years.
- China and the U.S. were the dominant countries in terms of publications, researchers, and institution collaboration in CADD.
- CADD, and AIDD (artificial intelligence-aided drug design), with the integration of artificial intelligence, have greatly facilitated lead compound discovery and optimization.

The development of new drugs involves a complex, slow, risky, and expensive system engineering process. In 1978, Stuart Marson and others from the University of California, with the participation of the computational chemist Todd Wipke, established the world’s first commercial company (MDL) dedicated to computer-aided drug design (CADD). These authors pioneered the development of a system for retrieving chemical reactions and databases. Since then, CADD has been widely used in the pharmaceutical industry to improve the efficiency of drug development pipelines. It has become one of the essential means for pharmaceutical companies to conduct drug research and development and has brought revolutionary upgrades to the field. In 1981, Fortune magazine published a cover story titled ‘The Next Industrial Revolution: Merck’s Computer-Aided Drug Design,’ which marked the beginning of the CADD craze sweeping through the entire pharmaceutical industry^1^.

**Figure 1:**
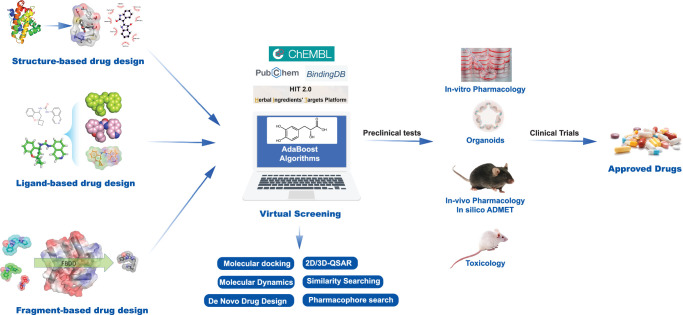
CADD (computer-aided drug design) flowchart. ADMET, absorption, distribution, metabolism, excretion, and toxicity; QSAR, quantitative–structure–activity relationship.

CADD is defined as all computer-assisted techniques used for discovering, designing, and optimizing compounds with desired structures and properties. CADD consists of various processes, including target identification, virtual screening of chemical libraries, optimization of lead compounds, and evaluation of potential toxicity, which are performed through computational simulation. This approach effectively reduces the number of compounds that need to be evaluated experimentally and aids in focusing resources on molecules with the highest potential to become drugs and alleviates the scale, time, and cost issues associated with traditional experimental methods^2,3^. Based on different principles of computer-aided drug screening, CADD includes structure-based drug design (SBDD) and ligand-based drug design (LBDD). SBDD methods include molecular docking, molecular dynamics (MD) simulation, and de novo drug design, while LBDD methods encompass the quantitative–structure–activity relationship (QSAR) model, similarity searching, and pharmacophore modeling^4^. Additionally, fragment-based drug design (FBDD) is a common drug design method involving fragment growing, fragment linking, and fragment merging techniques^5^. The drug design workflows of CADD and FBDD are shown in Figure 1. The ultimate goal of CADD is to virtually screen large compound databases to generate a set of hit compounds and lead compounds or to optimize known lead compounds, transforming bioactive compounds into suitable drugs through improvements in their physicochemical, drug, absorption, distribution, metabolism, excretion, toxicity (ADMET)/pharmacokinetic (PK) properties.

In recent years, there has been an explosive increase in the number of articles related to CADD. The rise of technologies such as structural biology, X-ray crystallography, genomics, and bioinformatics has also driven the development of CADD, making it a powerful tool for collaboration across various disciplines. Therefore, this study provides an overview based on bibliometrics, visually sorting out the developmental trajectory of the CADD field. By following a timeline, we analyze hot topics in CADD research and explore future research trends, showcasing the major contributions and existing problems in the development of CADD over the past 20 years. Thus, this review may serve as a reference for the development of CADD and the transformation of drug research and development models.

## Materials and methods

### Data source and literature retrieval

The literature data was sourced from the WOSCC database, with the retrieval period ranging from 2000 to 20 July 2023. Web of Science was chosen as the primary database for this study because of its comprehensive coverage of more than 12 000 scholarly journals and its frequent use in visual analytical studies^6–8^. Compared to other databases such as Scopus, Medline and PubMed, Web of Science provides the most comprehensive and reliable bibliometric analyses^9^. The search was conducted using the search query TS=(computer-aided drug design) AND (computer aided drug design).

### Data processing

#### Inclusion criteria

(1) Literature related to computer-aided drug design; (2) Literature published in English; (3) Types of literature include clinical trial research, experimental research, reviews, etc.; (4) Bibliographic information must be complete (including title, country, authors, keywords, and source).

#### Exclusion criteria

(1) Conference papers and newspapers; (2) Duplicated publications.

### Data normalization

The selected literature was exported in plain text format, with any embedded special characters removed. The Data Import/Export function within the CiteSpace software was then used to normalize the keywords, countries, and institutions associated with the retrieved literature.

### Methods and data analysis

We employed Excel software (version 16.67) for the analysis of literature publication volume. VOSviewer (version 1.6.18) was used for visual analysis of core authors and keywords in the literature^10^. RStudio software (version R-4.2.2) with the bibliometrix package was utilized for visual analysis of literature journals, countries, cited authors, and keyword clouds^11^. CiteSpace software (version 6.1.R3) was employed for the analysis of research institutions and cited literature^12^.

In this process, keywords were integrated, and synonyms were merged. The classification or categorization provided by the software itself is based on mainstream bibliometrics algorithms. The transformed dataset files were then analyzed using CiteSpace software for keyword and institution visualization. The centrality of keyword nodes was calculated concurrently. A higher centrality value indicates that the node plays a significant role in the visualized network. Additionally, in the author’s visual analysis, we analyzed based on Price’s law formula, where core authors have a publication count greater than *M*_p_ ( $M_{P}=0.749*\sqrt{N_{\mathrm{pmax}}}$ , with *M*_p_ being the minimum publication count for an author and *N*_pmax_ being the maximum number of papers produced by an author. Finally, in the keyword clustering analysis method, the LLR algorithm was applied to form a keyword clustering view. The LLR algorithm is an extraction algorithm provided by CiteSpace for cluster label extraction, and the software itself conducts cluster analysis. Modularity (*Q* value) and average silhouette width (*S* value) are used to evaluate the effectiveness of the clustering. Generally, a *Q* value >0.3 suggests significant cluster structure, while an *S* value >0.5 indicates reasonable clustering. This review complied with the PRISMA (Supplemental Digital Content 1, http://links.lww.com/JS9/C119; Supplemental Digital Content 2, http://links.lww.com/JS9/C120) (Reporting Items for Systematic Reviews and Meta-Analyses) and the AMSTAR (Supplemental Digital Content 3, http://links.lww.com/JS9/C121) (Assessing the Methodological Quality of Systematic Reviews) guidelines^13^.

## Results

### Literature retrieval and screening results

**Figure 2:**
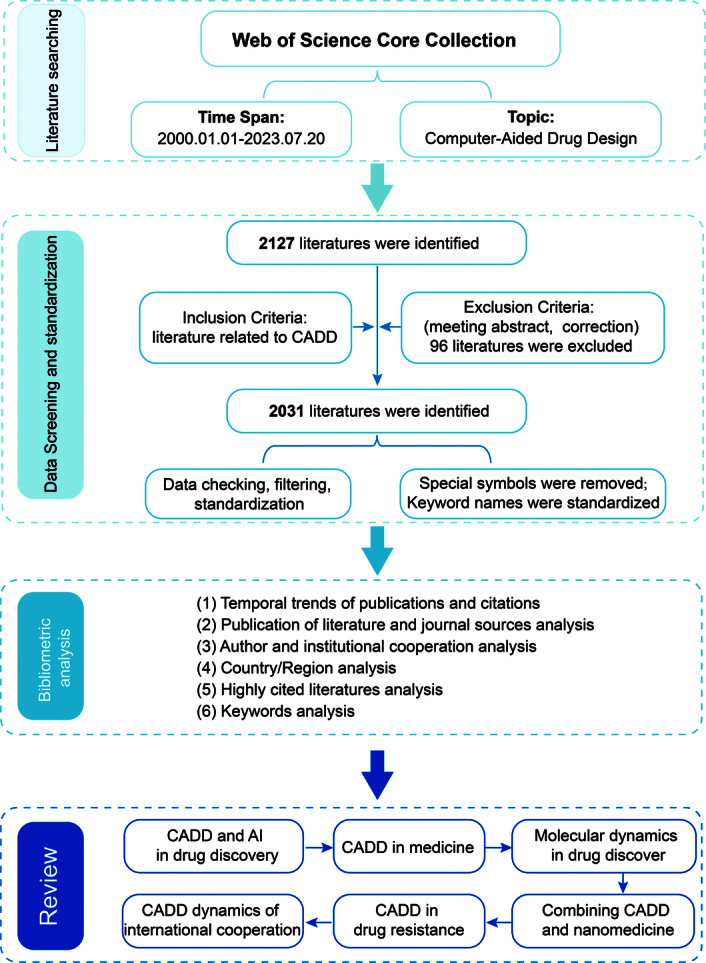
Literature data screening process and technology roadmap. CADD, computer-aided drug design.

A total of 2127 articles were retrieved through the search. After duplicate removal, screening, and review of the articles, 2031 articles were selected based on the inclusion and exclusion criteria. For the detailed steps, refer to Figure 2.

### Annual publication and journal source analysis of the literature

**Figure 3:**
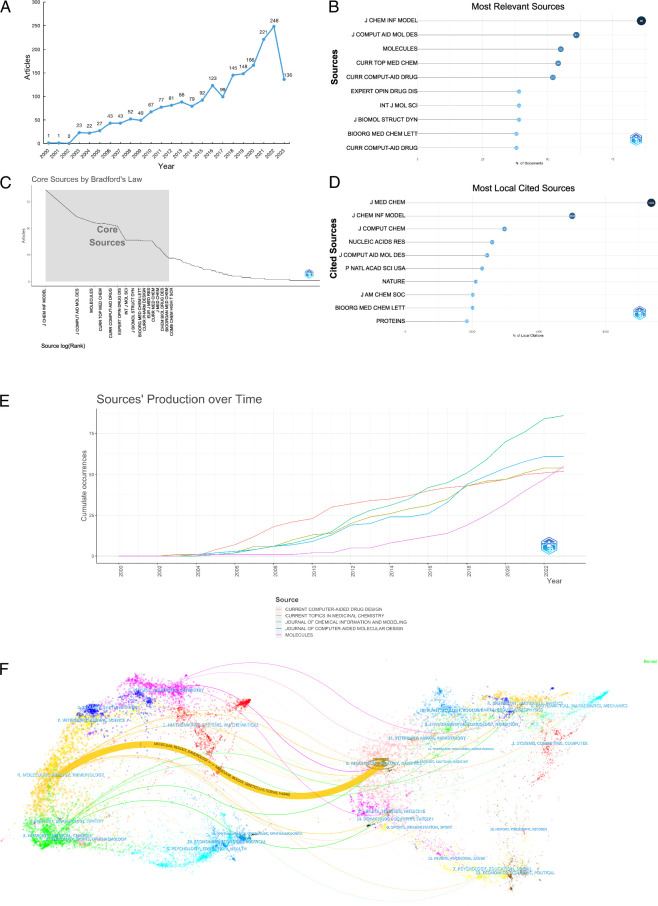
Visualization of publication quantity and journal sources. (A) Annual distribution of publication quantity; (B) Top 10 journals in terms of publication quantity; (C) Core journals determined according to Bradford’s law; (D) Top 10 journals cited in the publications; (E) Annual production trends of the top 5 journals in terms of publication quantity; (F) Dual map of journal citation relationships, with clustering of citing and cited journals.

The analysis of publication volume in the field of computer-aided drug design technology showed an overall upward trend, as illustrated in Figure 3A. Figure 3A shows that there was a significant decrease in the volume of publications in 2016–2017, which can be related to major international events at that time, such as in June 2016, when the UK referendum led to its decision to leave the European Union. This event triggered much uncertainty and turmoil, which had a definite impact on UK research and transnational collaboration. However, in 2017, the U.S. passed the ‘Future Act’ to elevate the development of artificial intelligence (AI) to the level of a national strategy and provide support for talent, investment, ethics, policy, law, and other aspects to create a strong foundation for the rapid development of AI. During the period 2017–2023, there was extensive use of AI, deep learning, and machine learning (ML), which led to a rapid increase in the number of publications, especially after the outbreak of COVID-19; this has led researchers worldwide to focus on the development of vaccines for major infectious diseases. A total of 561 journals published these articles. *Journal of Chemical Information Model* was the journal with the highest number of published articles, totaling 86, as shown in Figure 3B. The following 15 core journals were identified according to Bradford’s law, as shown in Figure 3C. The journal *J Chem Inf Model* focuses on the following areas: cheminformatics, molecular simulation and computational chemistry, molecular descriptors and drug design, and visualization of chemical information. The journal is dedicated to the application of methods and techniques such as informatics, computational chemistry and ML in chemistry. It covers all aspects of cheminformatics, including areas such as chemical data management, molecular simulation, drug design, and visualization of chemical information. Moreover, *J Chem Inf Model* welcomes descriptions and evaluations of new chemical databases or software tools. *Journal of Medicinal Chemistry* is the most cited journal in the field of computer-aided drug design technology, as shown in Figure 3D. *J Med Chem* is an important journal in the field of medicinal chemistry that focuses on the following areas: new drug discovery and development, drug design and chemical optimization, drug metabolism and pharmacokinetics, drug targets and biological activity. For readers or scholars in related fields, it is worthwhile to follow both journals.

As shown in Figure 3E, the *Current Computer-Aided Drug Design* journal led in publication volume before 2015, while after 2015, *J Chem Inf Model* saw rapid growth in publication volume and has since maintained its lead. The overlay of dual maps of journals demonstrates the citation relationship between the citing and cited journals, with clusters of citing journals on the left and clusters of cited journals on the right. As shown in Figure 3F, the orange pathway indicates that research from journals in the fields of molecular biology and immunology is most likely to be cited by journals in molecular biology, genetics, chemistry, materials, and physics. The pink pathway suggests that research from journals in chemistry, materials, physics, molecular biology, and genetics is most likely to be cited by journals in physics, materials, and chemistry.

### Visualization analysis of international collaboration

**Figure 4:**
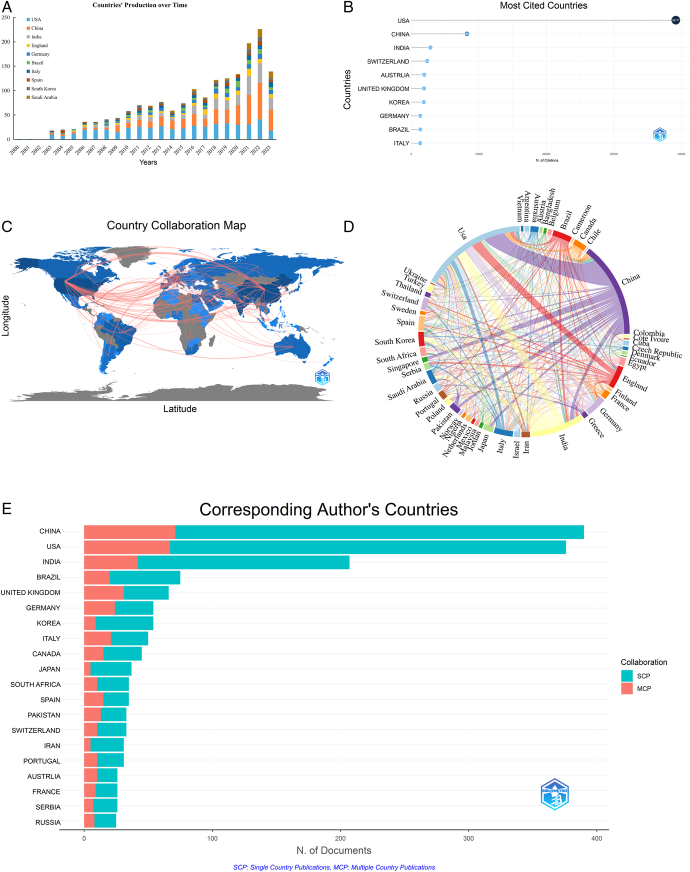
Visualization map of countries of origin of publications. (A) Distribution of publications by country; (B) Top 10 countries by citation count for publications; (C) Distribution map of collaboration between countries; (D) Chord diagram of collaboration distribution between countries; (E) Top 10 countries by number of corresponding authors.

Literature related to CADD has been published by authors from 99 countries. As shown in Figure 4A, the top 10 countries in terms of publication volume are the USA (490 articles), China (445 articles), India (249 articles), England (105 articles), Germany (94 articles), Brazil (88 articles), Italy (87 articles), Spain (68 articles), South Korea (66 articles), and Saudi Arabia (65 articles). As indicated in Figure 4B, the country with the most citations of its literature was the USA, followed by China. Figure 4C, D display the collaboration relationships in the CADD field between different countries; a thicker line between two countries suggests more collaboration between them. From this, we can infer that there is closer collaboration between the U.S. and China and other countries. The U.S. dominates both in terms of the impact of publications (ranked first in the number of citations) and the number of publications (ranked second), with the University of California and the University System of Maryland as the main publishers, as well as AstraZeneca International Pharmaceuticals, which is headquartered in the U.S. In China, the Chinese Academy of Sciences is the main issuing organization.

‘Single-Country Publications (SCP)’ refers to articles in which all the authors are from the same country, while ‘Multiple Country Publications (MCP)’ denotes articles written by authors from multiple countries, indicating international collaboration. Based on this, it is inferred that China has the most collaborative authors in this field from other countries, but there is still a larger proportion of collaboration within China itself, as shown in Figure 4E. As seen in the ranking of the number of publications by the corresponding author, eight of the top 20 countries belong to the Asia-Pacific region. The computer-aided drug discovery market in the Asia-Pacific region is anticipated to experience lucrative growth. This growth is mainly attributed to the increase in research and innovation by the healthcare firms that are operating in the region. Additionally, the number of patients suffering from multiple diseases, such as cardiovascular diseases (CVD) and diabetes, is increasing dramatically, particularly in China and India, and may also have a positive impact on the market throughout the forecast period.

### Visualization analysis of author and institution collaboration

**Figure 5:**
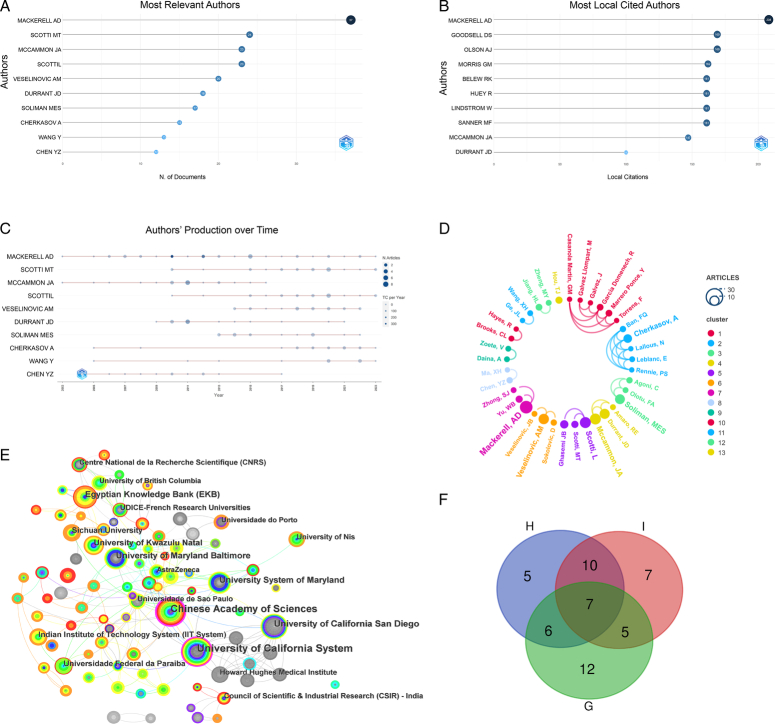
Visualization map of authors and institutions. (A) Top 10 authors by number of publications; (B) Top 10 most cited authors by publication; (C) Annual publication trend of the top 10 authors (each point represents an author’s publications at a particular point in time, the size of the point represents the number of publications, and the color shade of the point represents the number of citations); (D) Network diagram of collaboration among authors (the same color represents a collaborative team); (E) Distribution map of collaboration among institutions (Larger font sizes mean more articles, and thicker lines mean closer co-operation); (F) Venn diagram of authors.

The visualization of authors in the 2031 articles included a total of 8836 authors. The author with the most published articles is MacKerell AD from the University of Maryland, with 37 articles. The changes in publication volume and annual publication volume for the top 10 authors are shown in Figure 5A and C. The author with the most cited articles is also MacKerell AD from the University of Maryland, with 208 citations, as shown in Figure 5B. According to Price’s Law formula, the *M*_p_ is determined to be 3.96; therefore, authors with a publication volume of ≥4 articles are defined as core authors in this study, resulting in 28 core authors. As shown in Figure 5D, each node represents an author, and the size of the node indicates the volume of articles published by the author. More connections between nodes suggest closer collaboration between authors, and different colors represent relationships between different collaborative groups. There are five closely collaborating academic teams in this field, including those led by MacKerell AD, Veselinovic AM, Scotti L, Mccammon JA, Soliman MES, and Cherkasov A. Alexander D MacKerell Jr., from the Center for Computer-Aided Drug Design, Department of Pharmaceutical Sciences, University of Maryland School of Pharmacy, was the author with the highest level of collaboration, working closely with Erik P H de Leeuw from the Department of Molecular Biology and Marie Hanscom from the Anesthesiology Research Center in the same school; these researchers complemented each other in their collaborations through their respective disciplinary strengths, resulting in the conformational exploration of a small-molecule Lipid II inhibitor and the design of a novel neuroprotective agent^14^. Artem Cherkasov from the Vancouver Prostate Centre, Columbia University, Canada, is engaged in an international collaboration with authors Ayse Derya Cavga, Turkey, and Mark R. Flory, USA, who work together on prostate cancer research^15^.

The top 10 institutions by publication number are listed in Table 1. The University of California System, with 67 articles, is the institution with the most publications. Betweenness centrality (BC) represents the importance of a node in the network. In the graph, each node represents an institution, a larger text indicates a greater volume of publications from an institution, and lines between nodes represent collaborative relationships between institutions. The institution with the highest BC is the Chinese Academy of Sciences. As shown in Figure 5E, there is close collaboration among the University of California System, the Chinese Academy of Sciences, and the Egyptian Knowledge Bank (EKB), among others.

**Table 1:** Top 10 institutions in terms of publications (left) and top 10 institutions in terms of centrality (right).

Rank	Institution	Publications	Rank	Institution	BC
1	University of California	67	1	Chinese Academy of Sciences	0.23
2	Chinese Academy of Sciences	56	2	University of California	0.18
3	University of California San Diego	36	3	Sichuan University	0.09
4	Egyptian Knowledge Bank (EKB)	32	4	Egyptian Knowledge Bank (EKB)	0.08
5	University of Maryland	31	5	Harvard University	0.08
6	University of Maryland Baltimore	31	6	Peking University	0.07
7	University of Kwazulu Natal	27	7	University of British Columbia	0.06
8	Sichuan University	22	8	Massachusetts Institute of Technology (MIT)	0.06
9	Indian Institute of Technology System (IIT System)	22	9	Indian Institute of Technology System (IIT System)	0.05
10	Universidade Federal da Paraiba	21	10	Centre National de la Recherche Scientifique (CNRS)	0.05

BC, betweenness centrality.

Performing an analysis of core authors, frequently collaborating authors, and highly cited authors in this field helps to capture the key content of the current research. Hence, we have used ‘H’ to designate core authors, ‘G’ to designate the top 30 authors by collaboration strength, and ‘I’ to designate the top 30 authors by citation count. Through analysis with a Venn diagram (Fig. 5F), we found that there are 7 authors common to categories H, G, and I. There are 10 authors common to I and H, and 6 authors common to H and G. There are 5 authors who are only in category H, 7 who are only in category I, and 12 who are only in category G. These 52 authors have made significant contributions to the development of this field, and their important research findings are displayed in Table 2. Details on the author data are shown in Supplementary Table S1, Supplemental Digital Content 4, http://links.lww.com/JS9/C122.

**Table 2:** The detailed information of the core authors, top 30 authors by collaboration strength, and top 30 authors by citation.

Relationship with I, H, and G	Author	Publications	Cooperation intensity	Cited index	Active year	Institute/country	Main contributions/references
IHG	Mackerell AD	28	96	30.12	2013	University of Maryland, USA	ERK inhibitors^16^, Anti-inflammatory compound UM101^17^, Antitumor compounds targeting BCL6^18^, Compounds for repairing DNA^19^
	Zhong Zhijun	6	33	8.26	2007	University of Maryland, USA	Heme oxygenase inhibitor^20^
	Hou Tingjun	9	24	8.01	2018	Zhejiang University, China	VGSC-DB(VGSC-DB(zju.edu.cn))^21^
	Yu Wenbo	9	22	7.47	2017	University of Maryland, USA	Antibacterial compounds BAS00127538^22^, Inhibitors against melanoma S100B^23^
	Jiang Hualiang	8	48	7.16	2013	Shanghai Institute of Materia Medica, China	Anti SARS-CoV-2 compound ebselen^24^, IPMF scoring function^25^
	Zheng Mingyue	6	39	5.46	2013	Shanghai Institute of Materia Medica, China	wPMF^26^, MOSFOM^27^
	Wang Xinhui	6	44	3.48	2020	The First Bethune Hospital of Jilin University, China	PARP inhibitor^28^, CD13 inhibitor^29^
IH	Vanommeslaeghe	4	5	78.09	2012	University of Maryland, USA	CHARMM general force field^30^, Automation of CGenFF^31^
	Raman E Prabhu	4	13	24.17	2014	University of Maryland, USA	Mapping functional group free energy patterns at protein occluded sites^32^, SILCS Pharm protocol^33^
	Wang Junmei	7	10	16.20	2018	University of Pittsburgh, USA	Incorporating structural similarity into a scoring function^34^, Identification of possible drug treatment of COVID-19^35^
	Xu Lei	6	8	5.39	2020	Jiangsu University of Technology, China	Combining SALSTM and GAT methods to predict molecular properties^36^, PDE5 inhibitor^37^
	Li Dan	7	13	5.27	2018	Zhejiang University, China	VEGFA inhibitor^38^, PPT/PPa-NPs^39^
	Wang Tao	5	1	5.06	2018	Deakin University, Geelong, Australia	Modify palustrin-2LTb^40^
	Lakkaraju Sirish Kaushik	4	13	4.64	2016	University of Maryland, USA	SILCS hot spot method^41^
	Wang Wei	4	3	3.76	2016	Dalian University of Technology, China	Novel proteasome inhibitors^42^, Anti-breast cancer compounds SP141^43^
	Chen Xi	4	14	3.75	2013	University of Maryland, USA	MGluR1 negative allosteric regulator found in traditional Chinese medicine^44^
	Wang Jian	4	5	3.43	2017	Shenyang Pharmaceutical University, China	PAK1 allosteric inhibitor^45^, MIEC-SVM docking method^46^
HG	Ge Junliang	5	40	2.99	2020	Jilin University, China	C-MET targeted drugs^47^, C-Myc inhibitor^48^
	Xue Fengtian	4	19	2.88	2015	University of Maryland, USA	Antibacterial compounds targeting HemOs^49^, Compound 22 for the treatment of traumatic brain injury^14^
	Wu Bo	4	34	2.85	2020	Jilin University, China	Antitumor drug AKT1 inhibitor^50^
	Shen Jingkang	4	28	1.99	2012	Shanghai Institute of Materia Medica	Developing a target-specific method for kinase–ligand interactions^51^
	Xiong Bing	4	28	1.99	2012	Shanghai Institute of Materia Medica, China	Anticancer compound HSP90 inhibitor^52^
	Li Weihang	4	29	1.46	2020	Jilin University, China	Compound MGMT targeted drugs^53^
IG	Zhu Xiao	3	29	5.02	2008	University of Maryland, USA	Non-toxic targeted BCL6 anti-tumor compound^18^
	Luo Xiaomin	3	28	4.39	2016	Shanghai Institute of Materia Medica, China	Artificial intelligence in drug design^54^
	Coop Andrew	2	16	4.35	2010	University of Maryland, USA	Introduction to ligand based drug design^55^
	Chen Kaixian	2	21	3.88	2018	Shanghai Institute of Materia Medica, China	New strong BRD4 inhibitors^56^
	Xing Jing	2	21	3.88	2018.5	Shanghai Institute of Materia Medica, China	Artificial intelligence in drug design^54^
I	Fang Lei	3	8	4.12	2017	University of Maryland, USA	Mixed nano micelle eye drops with valine ligands^57^
	Tian Sheng	2	4	3.83	2018	Soochow University, China	Anti-inflammatory compound IRAK4 inhibitor^58^
	Wang Jin	3	2	3.61	2016	Soochow University, China	Ras protein hydrolysis intermediate conformation inhibitors^59^
	Wang Meng	2	1	3.37	2022	Heilongjiang University of Chinese Medicine, China	Identify the potential mechanism of celastrol against osteoarthritis^60^
	Fu Zunyun	1	15	3.36	2018	Shanghai Institute of Materia Medica, China	Artificial intelligence in drug design^54^
	Li Fei	1	15	3.36	2018	Shanghai Institute of Materia Medica, China	Artificial intelligence in drug design^54^
	Li Xutong	1	15	3.36	2018	Shanghai Institute of Materia Medica, China	Artificial intelligence in drug design^54^
H	Shapiro Paul	4	6	2.05	2009	University of Maryland, USA	Targeting p38 α anti-inflammatory compound UM101^17^, A non-ATP interfering MAP kinase inhibitor^61^
	Li Honglin	5	15	1.70	2015	East China University of Science and Technology, China	Drug Discovery and Design Platform iDrug (http://lilab.ecust.edu.cn/idrug)^62^, PTID (http://lilab.ecust.edu.cn/ptid/)^63^
	Wang Ying	4	1	1.66	2014	Shenyang Pharmaceutical University, China	Revealing anti-diabetes mechanisms of natural product^64^, Provide a method to detect non-specific interactions between ligands and cell membranes^65^
	Li Wei	4	5	1.62	2016	Nanjing University of Chinese Medicine, China	Selective human butyrylcholinesterase inhibitors for anti-AD^66^, Optimize PAK1 inhibitors^45^
	Xu Ping	4	1	1.38	2015	Peking University, China	Potential proteasome inhibitors^67^, Activating AKT to inhibit JNK^68^
G	Melnick Ari	2	17	2.94	2014	Cornell University, USA	New BCL6 inhibitor^69^, Targeting BCL6 anti-tumor compounds^18^
	Zhong Sheng	3	29	2.79	2020	Sun Yat-sen University, China	PARP inhibitor^28^
	Liu Wei	3	21	2.67	2017	Case Western Reserve University, USA	SHP2 inhibitor^70^, Targeting SHP2 for the treatment of PTPN11-associated malignancies^71^
	Lopes Pedro e. m.	2	19	2.65	2014	University of Maryland, USA	Novel anti-tumor Notch inhibitors^72^
	Yang Wenzhuo	3	31	2.45	2021	Jilin University, China	C-MET targeted drugs^43^
	Zhang Zhiyun	3	31	2.45	2021	Jilin University, China	Optimizing antitumor drug AKT1 inhibitors^50^
	Mou Yonggao	2	25	2.31	2021	Sun Yat-sen University, China	C-MET targeted drugs^28^
	Wang Hongyu	2	25	2.31	2021	Jilin University, China	Optimizing antitumor drug AKT1 inhibitors^50^
	Astudillo Luisana	1	16	1.90	2016	University of Miami, USA	Novel anti-tumor Notch inhibitors^72^
	Li Yanlian	3	21	1.49	2012	Shanghai Institute of Materia Medica, China	Anticancer compound HSP90 inhibitor^72^
	Li Jing	3	25	1.02	2013	Lanzhou University, China	Novel γ-aminobutyric acid derivatives^73^, Phosphodiesterase 4B inhibitor^74^
	Liang Li	2	16	0.64	2018	Xuzhou Medical University, China	Applications of molecular simulation in the discovery of antituberculosis drugs^75^, Dihydromyricetin can be used as a novel antibacterial lead compound^76^

### Analysis of the cited literature

**Figure 6:**
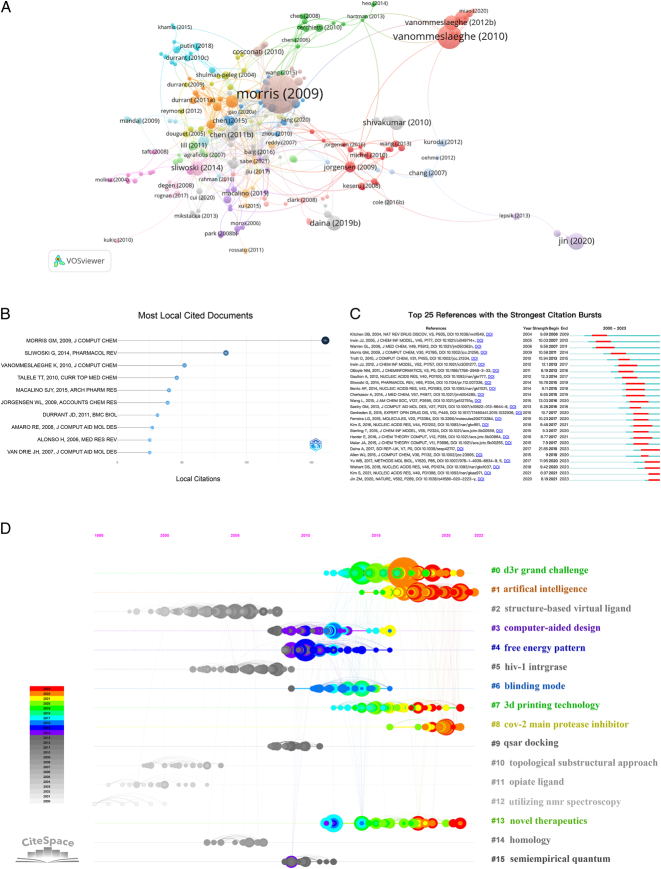
Visualization map of cited literature. (A) Thematic coupling analysis of research literature (the node size indicates the number of cited documents; the connecting line indicates a collaborative relationship (cited by the same document); as for the color, it is the cited authors clustering); (B) Top 10 cited articles; (C) emergent analysis of cited literature; (D) Timeline analysis of cited literature.

As shown in Figure 6A, the thematic coupling analysis of the research literature resulted in eight clusters: the purple cluster includes most of the articles about protein ligands; the blue cluster focuses on themes related to computational molecular modeling experiments; the red cluster primarily centers on drug targets; the gray cluster mainly focuses on small-molecule protein targets; the orange cluster emphasizes the design of new drugs for multiligand receptor complexes; the yellow cluster encompasses most of the articles related to protein databases; the pink cluster focuses on molecular docking; and the green cluster primarily deals with traditional Chinese medicine computer-aided drug screening databases. According to Figure 6B, the three most cited articles are Morris GM, 2009, and *J Comput Chem* (cited 161 times), which mainly introduce the use of the AutoDock4 program for automated docking^76^. Sliwoski G, 2014, *Pharmacol Rev* (cited 84 times) introduces the theories behind CADD methods and showcases their latest applications^77^. Vanommeslaeghe K, 2010, *J Comput Chem* (cited 52 times). Among the top 10 most cited articles, five involve molecular docking in drug design.

Citation bursts refer to a sudden increase in citation frequency within a short time frame, highlighting research hotspots within specific periods and indicating shifts in research focus over time. It can also be used to understand the dynamics and trends of research hotspots. Figure 6C shows the top 25 cited references associated with citation emergence, most of which focus on molecular docking techniques and methods (Morris GM, Allen WJ, Yu W), database platform development (Bento AP, Kim S, Daina A), drug design and vaccine development (Jin Z, Ferreira LG), etc. A cluster silhouette value (*S*) of 0.8587 and a modularity value (*Q*) of 0.8577 indicate that the clustering is significant and reliable. The clustering of the cited literature is shown in Figure 6D. Clusters 2, 3, 6, 7, 9, 10, 11, 12, and 14 include research related to CADD technology and molecular docking; clusters 0, 1, 4, 8, 13, and 15 indicate that AI, protease inhibitors, and innovative therapies are actively pursued hot research topics; and cluster 5 is related to HIV disease research.

### Visualization analysis of keywords

**Figure 7:**
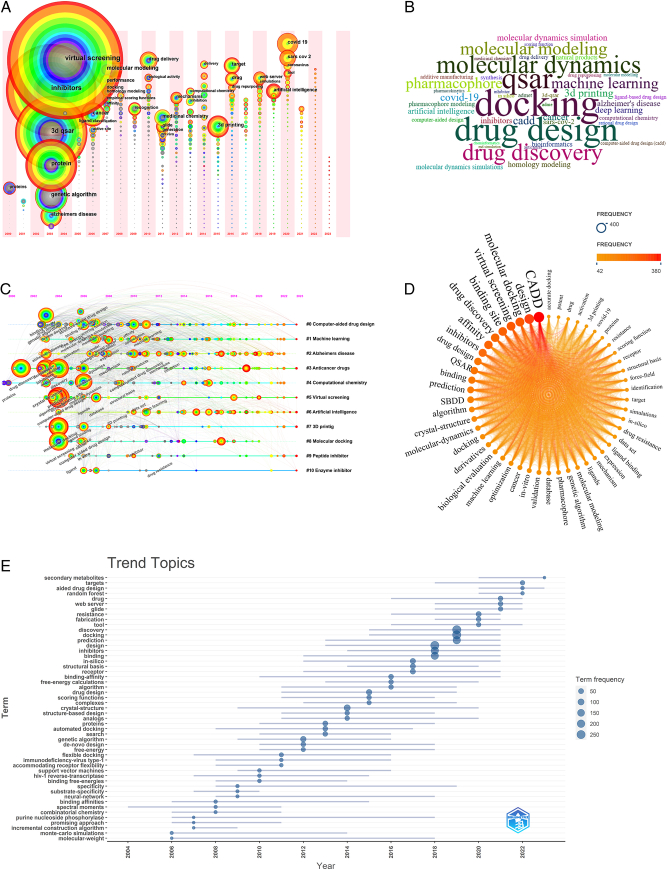
Keyword visualization analysis. (A) Keyword timeline; (B) Word clouds of keywords plus in local clusters; (C) Keyword time zones; (D) Keyword frequency distribution; (E) Trend topics of research of CADD from 2004 to 2022.

Keywords represent the core content and research themes of the literature. A keyword research hotspot time zone map can display the distribution and changes in keywords within a theme or field over different periods, helping us understand the hotspot themes of CADD research and their evolution over time from a temporal perspective. Figure 7A illustrates that over nearly 20 years, the field of CADD has experienced three stages. In the first stage (2000–2007), development was in its initial phase, with high-frequency keywords such as “virtual screening”, “inhibitors”, “3D QSAR”, and “protein” appearing prominently. During this stage, the main diseases of interest were Alzheimer’s disease and cancer. In the second stage (2008–2016), “drug delivery”, “biological activity”, and “3D printing” became new focal points. In the third stage (2017–2022), the introduction of “artificial intelligence” facilitated the development of CADD, with drug design for COVID-19 becoming a research focus in 2020. Additionally, we analyzed the high-frequency keywords within these 2031 articles. There are 52 keywords that appear more than 40 times, as depicted in Figure 7B, with larger font sizes representing higher occurrence frequencies.

The keyword cluster network map is shown in Figure 7C. With a cluster silhouette value (*S*) of 0.6782 and a modularity value (*Q*) of 0.4226, the clustering was significant and reliable. The visualization of keywords resulted in 10 cluster tags, which, to some extent, display the general research directions and dynamic evolution process in this field. Among them, #0 (CADD), #1 (machine learning), #5 (virtual screening), and #6 (artificial intelligence) were consistent themes throughout. Clusters #0 (CADD) and #8 (molecular docking) show close connections with the other clusters. Figure 7D displays the frequency distribution of keywords, with high-frequency words including CADD, design, molecular docking, and virtual screening, which is consistent with the results shown in Figure 7B. The top 25 burst keywords are shown in @@Supplementary Figure S1. The keyword trend distribution revealed that from 2006 to 2022, 51 leading topics were researched more than 50 times, among which discovery, docking, prediction, etc., were the most influential topics, as shown in Figure 7E.

## Discussion

**Figure 8:**
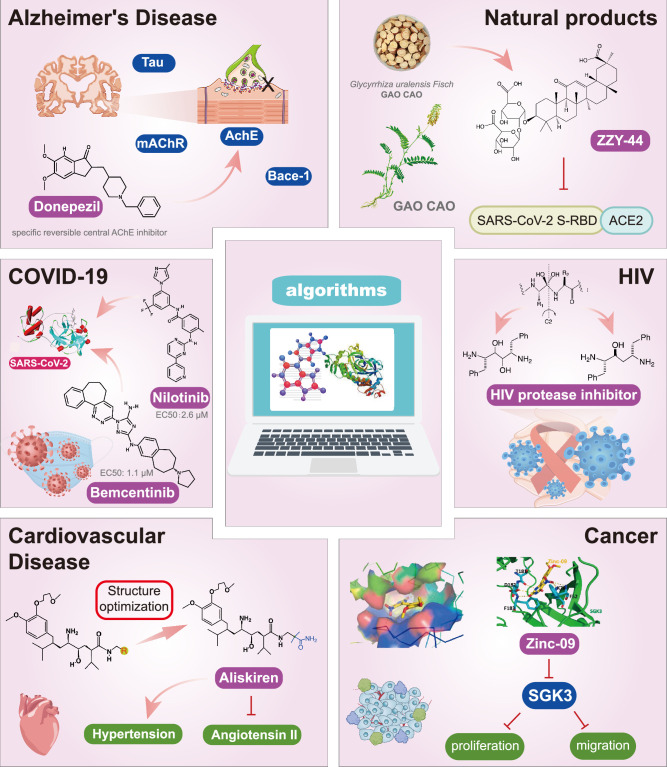
Applications of CADD in medical pharmacology. ACE2, angiotensin-converting enzyme 2; CADD, computer-aided drug design; COVID-19, coronavirus disease 2019; RBD, receptor-binding domain; SARS-CoV-2, severe acute respiratory syndrome coronavirus 2.

In recent years, the major hot topics in CADD have been distributed in fields such as medicine, agriculture, botany, materials science, environmental science, nutrition science, and cosmetics design^78–84^. Among these topics, the field of medicine has been the subject of the most related research, where CADD has been widely used for exploring therapeutic drugs for diseases such as Alzheimer’s disease, CVD, HIV, COVID-19, and cancer. CADD has also been used in drug discovery and design, identifying disease targets, researching drug resistance, and studying traditional Chinese medicine (TCM) (as shown in Fig. 8 and Table 3), as it has accelerated new drug development. In 1995, the importance of CADD in drug development was validated when dorzolamide, the first carbonic anhydrase inhibitor drug developed using CADD technology for treating glaucoma, successfully entered the market. Furthermore, CADD has played a part in the successful development of various other marketed drugs, including the angiotensin-converting enzyme inhibitor captopril, the HIV protease inhibitor saquinavir, and the fibrinogen antagonist tirofiban. The drugs developed based on the SBDD, LBDD, and FBDD technologies are shown in Tables 4–6. Since then, structure-driven CADD has continued to be an effective means for drug screening. According to the bibliometric analysis of CADD mentioned above, several frontiers and challenges are listed:

**Table 3:** Hot topics of CADD research in the medical field.

Hot topics	Research type	Optimization/new discoveries	Application	Contributor (year)
Drug design	Kinase inhibitors	Non-ATP-dependent MAP inhibitors	Inflammatory diseases, cancer, neurodegenerative diseases	Chad N Hancock (2006)^61^
	HIV-1 reverse transcriptase inhibitor	Optimization of affinity for binding of wild-type RT, tolerance to viral mutations	HIV	William L Jorgensen (2006)^85^
	Thrombin inhibitor	Compounds 14a–h exhibit greater anticoagulant activity than argatroban	Anticoagulation	Meilin Li (2015)^86^
	Drugs for autoimmune diseases	Significantly inhibits B-cell maturation antigen (BCMA) interaction with B-lymphocyte stimulating factors	Rheumatoid arthritis, lupus	Xiafei Hao (2016)^87^
	Drugs for depression	High affinity for HSPB8	Depression, neurodegenerative diseases	Sheikh Arslan Sehgal (2016)^88^
	GPR119 agonist	Optimizing toxicity risk	Type 2 diabetes	Fereshteh Shiri (2017)^89^
	Drugs for neurodegenerative diseases	High absorption, low toxicity	Alzheimer’s disease, neurodegenerative diseases	Oluwafemi Adeleke Ojo (2021)^90^
	SARS-CoV PLpro inhibitor GRL0617	Papain-like protease (PLpro)	COVID-19	Xiaopan Gao (2021)^91^
Target prediction	COVID-19 new target discovery	Glu166	COVID-19	Souvik Banerjee (2021)^92^
	Drug target prediction method	LigTMap	Small molecule compound target prediction	Faraz Shaikh (2021)^93^
	Target binding conformational tools	PacDOCK	Analyzing molecular docking results	Jacopo Carbone (2022)^94^
	New targets for cancer metabolic pathways	HPKM2	Cancer	Ludovico Pipitò (2023)^95^
Drug resistance	Combination drugs to reduce resistance	Combination of vancomycin and oseltamivir for the treatment of IAVs	Influenza A virus (IAV)	Yajun Sheng (2013)^96^
	HIV drug resistance prediction	Optimize ADME, reduce toxicity	HIV	Lucianna Helene Santos (2015)^97^
	Multi-target design reduces drug resistance	Preference for combined or overlapping pharmacophores	Drug resistance issue	Alan Talevi (2016)^98^
	SGK3 drug resistance design	Zinc-09 compounds inhibit proliferation and migration of ER+ breast cancer cells both in vivo and in vitro	Breast cancer	Duanfang Zhou (2022)^99^
	Human Cancer Resistance Protein (BCRP)	FLAPpharm method	Drug resistance	Laura Goracci (2023)^100^
Personalized medicine	DNA topoisomerase	Selective inhibitory activity against the α-subtype	Cancer	Malgorzata N Drwal (2014)^101^
	Individualized drug delivery program design	Childhood Tuberculosis Portfolio Programme	Tubercular disease	Shashikant Srivastava (2016)^102^
	Customization of drug dosage	3D printing technology	Precision dosage	Anish Chandekar (2019)^103^
	Intelligent drug delivery carriers	Nanotechnology	Precision drug delivery	Imran Ali (2020)^104^
TCM	Chemotherapy drug potentiation	Glycyrrhizic acid, lycopene C, comedic acid, and salvinorin B as potential Ku86 inhibitors	Complementary chemotherapy	Mao-Feng Sun (2011)^105^
	Database of active ingredients in TCM	TCM Database	Small molecule screening of TCM	Calvin Yu-Chian Chen (2011)^106^
	Screening of active ingredients in TCM	ZZY-44 is an effective, non-toxic, broad-spectrum anticoronavirus molecule	Antiviral herbal compounds	Shaopeng Yu (2021)^107^
	Analysis of the efficacy mechanism of TCM	Predicting 40 anticoagulation targets	Anticoagulation	Jia Lin (2022)^108^
	Quality assessment of TCM	Q-Marker: atractyloside, atractylenolide I and atractylenolide III	Evaluating the medicinal quality of atractylodes macrocephala	Yanyun Zhao (2023)^109^

CADD, computer-aided drug design.

**Table 4:** List of clinically approved drugs discovered by SBDD.

Drug	Target	Structure	Indication	Approved in year	Clinical trial ID	Company	References
Abrocitinib	JAK2/FLT3	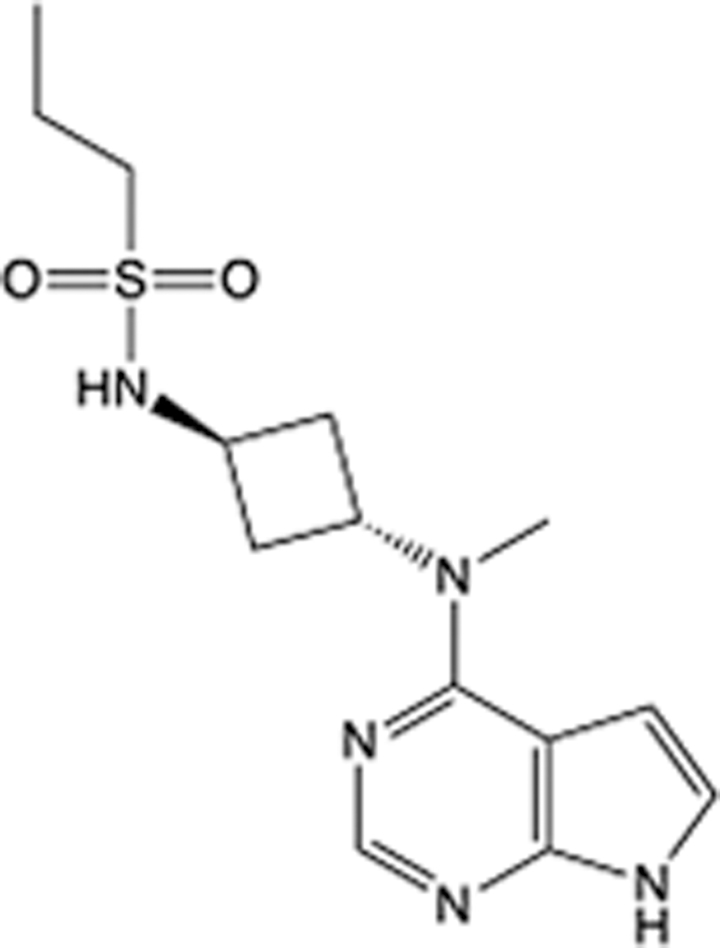	Moderate-to-severe atopic dermatitis	2022	NCT05602207	^Pfizer^	^110^
Tepotinib	c-Met	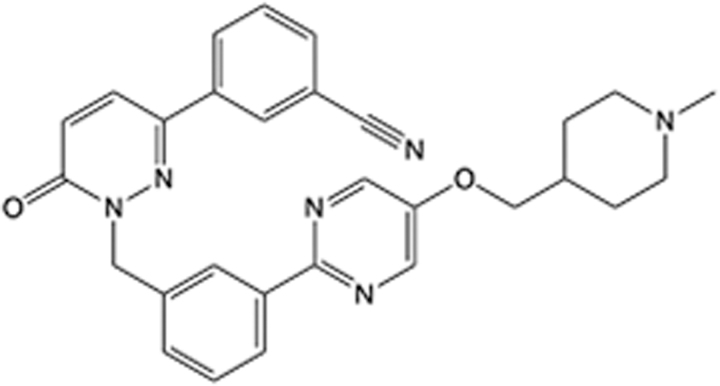	Non-small cell lung cancer	2022	NCT02864992	^Merck^	^111^
Alpelisib	PI3Kα	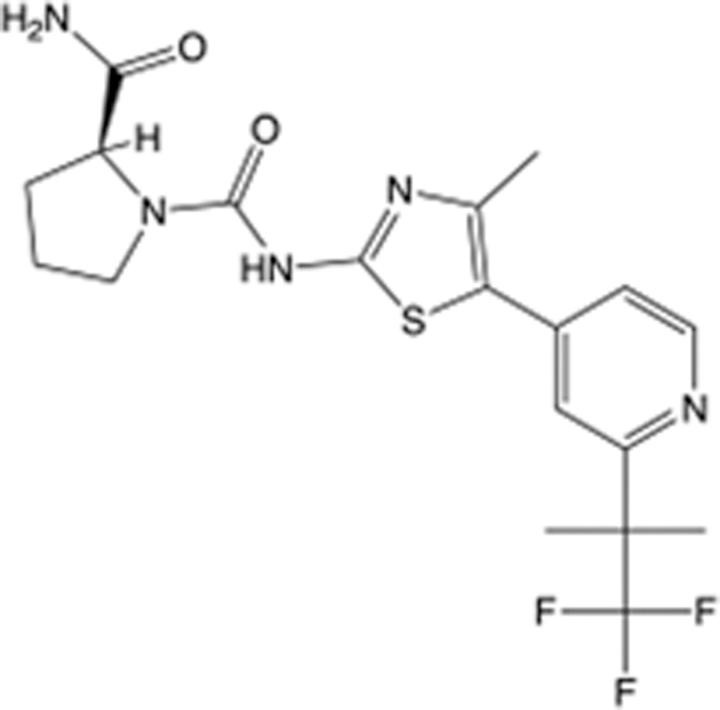	Breast cancer	2019	NCT02437318	^Novartis^	^112^
Boceprevir	NS3/4A serine protease	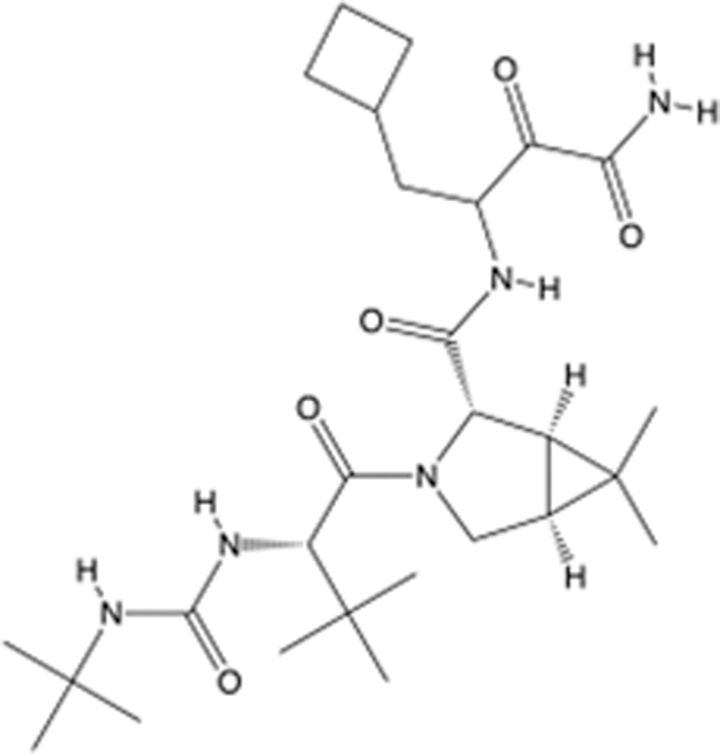	Hepatitis C, chronic	2011	NCT00705432	^Schering-Plough^	^113^
Crizotinib	c-MET, ALK	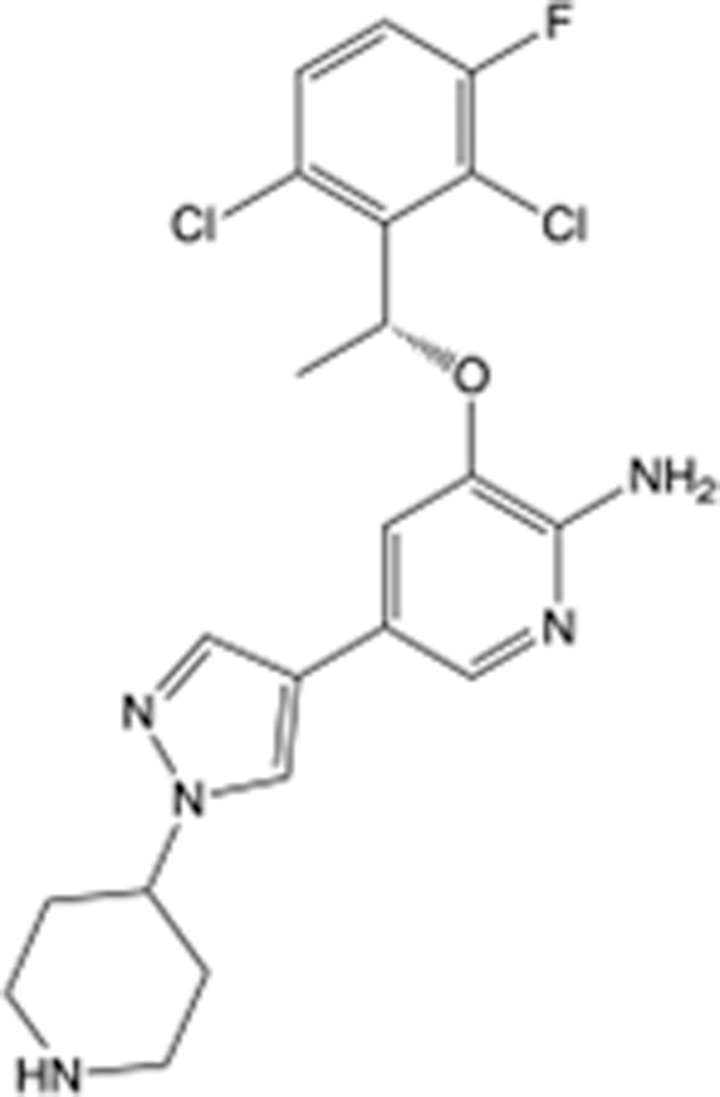	Advanced cancers	2011	NCT01744652	^Pfizer^	^114^
Aliskiren	Renin	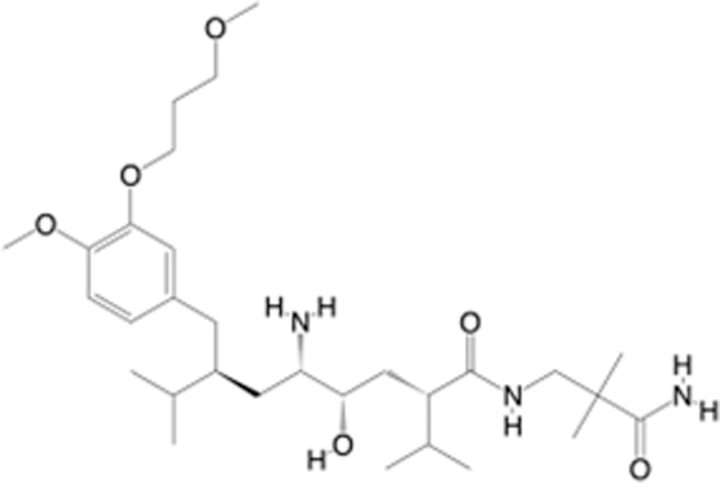	Hypertension	2007	NCT00311012	^Novartis^	^115^
Sorafenib	Serine/threonine and tyrosine kinase	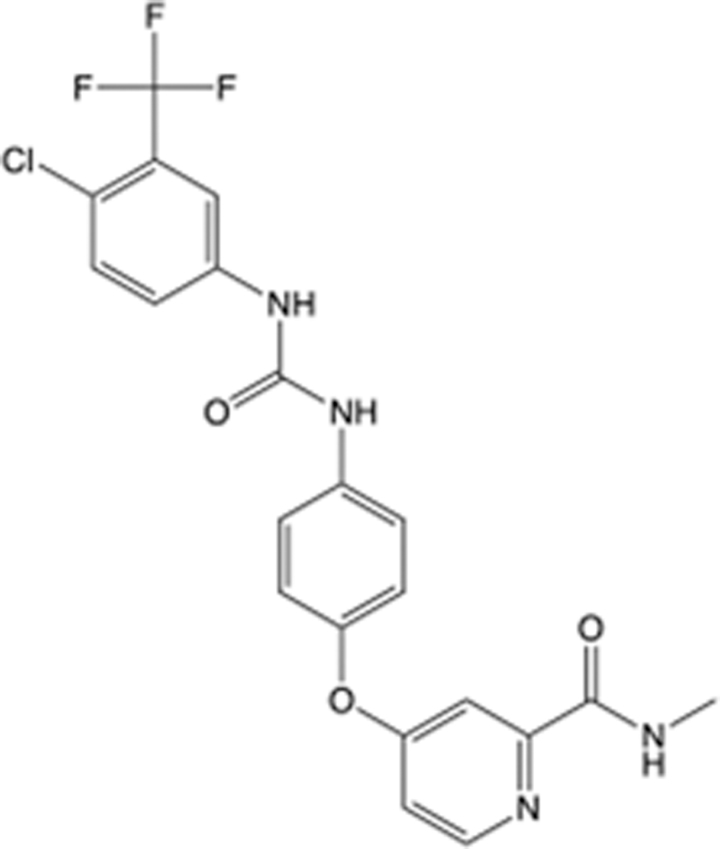	Advanced hepatocellular carcinoma	2005	NCT00105443	^Bayer^	^116^
Erlotinib	EGFR	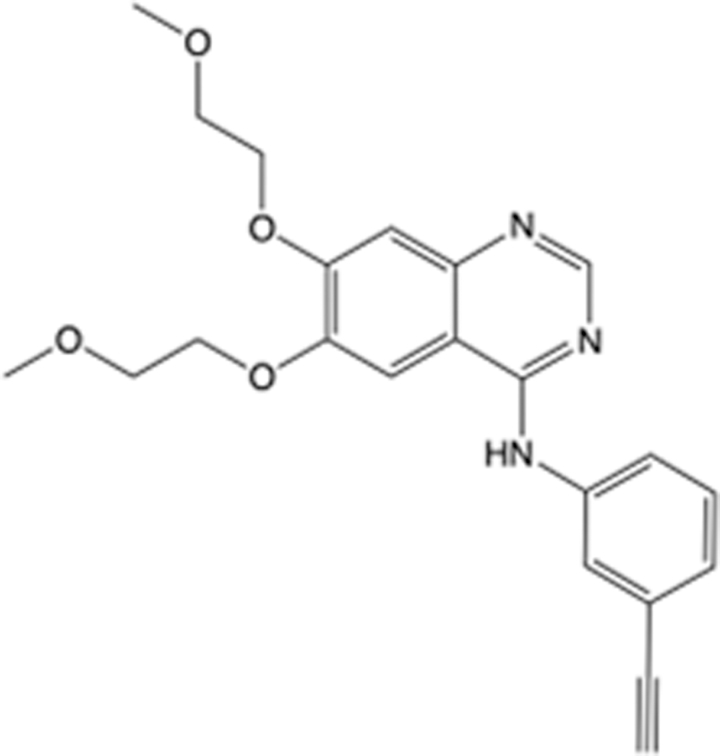	Non-small cell lung cancer	2004	NCT01287754	^Roche^	^117^
Gefitinib	EGFR	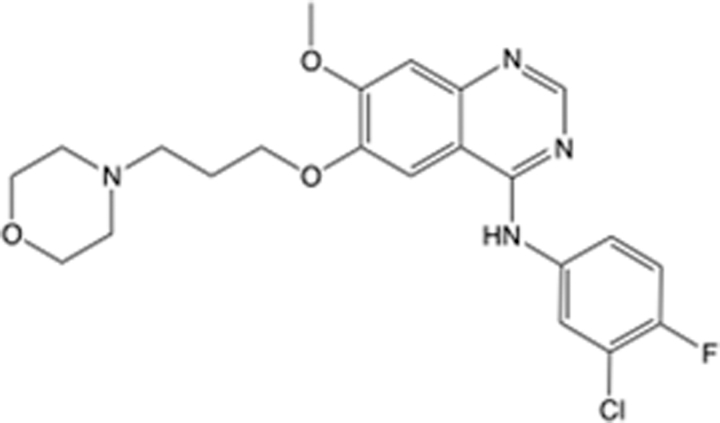	Non-small cell lung cancer	2003	NCT01203917	^AstraZeneca^	^118^
Oseltamivir	Neuraminidase	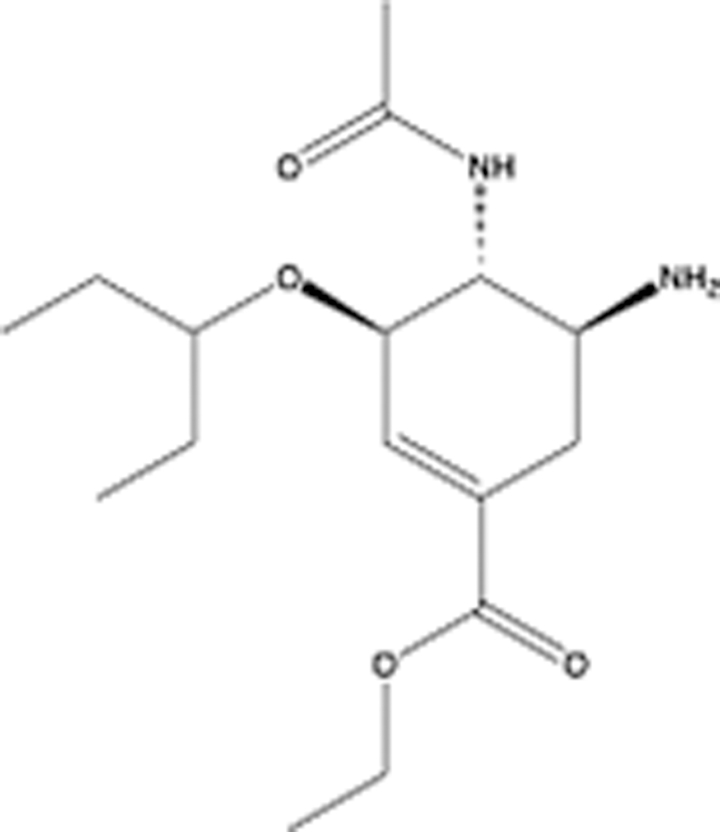	Influenza	1999	NCT00304434	^Roche^	^119,120^
Zanamivir	Neuraminidase	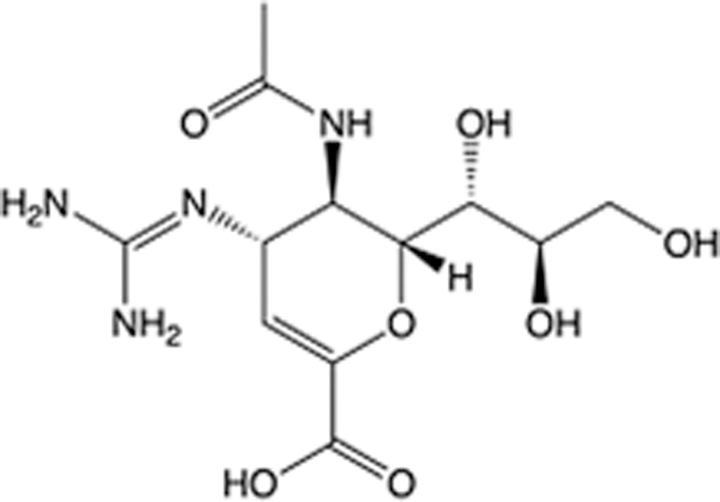	Influenza	1999	NCT01527110	^Biota^	^120,121^
Tirofiban	Gp IIb/IIIa, fibrinogen	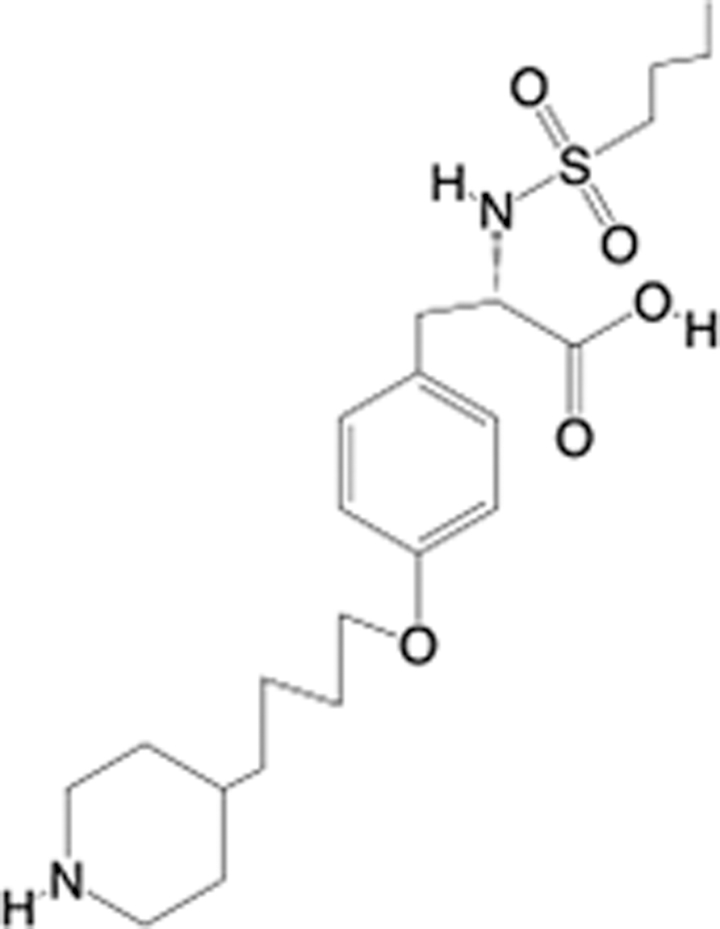	Acute coronary syndrome	1998	NCT00790387	^Merck^	^122^
Dorzolamide	Carbonic anhydrase II	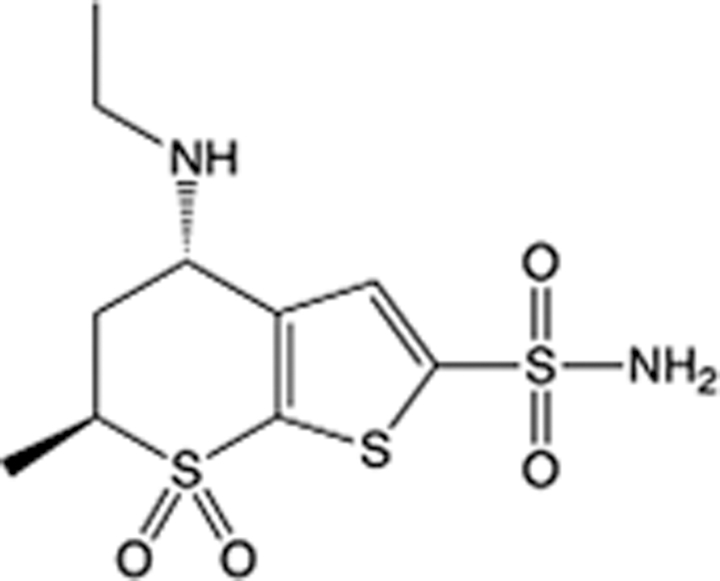	Glaucoma	1995	NCT00546286	^Mecrk/Dohme^	^123^
Captopril	ACE1	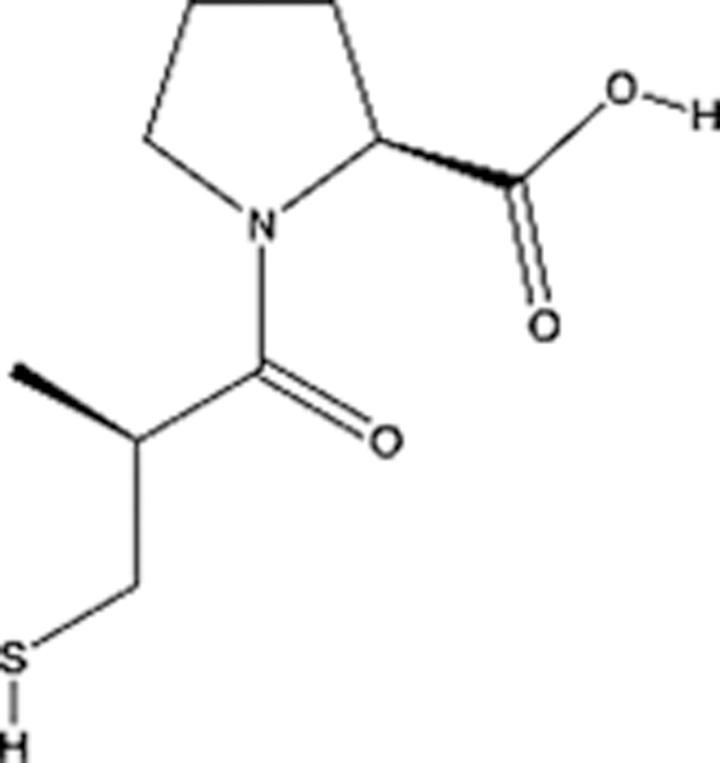	Hypertension	1981	NCT00240656	^Bristol-Myers Squibb^	^124^
AT7519	CDK2	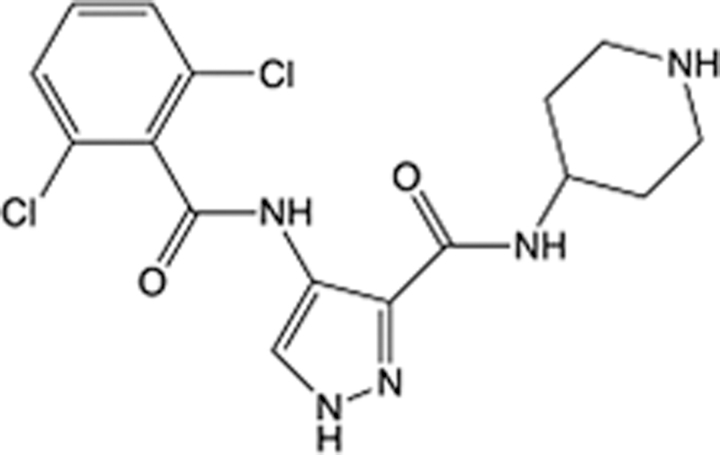	Chronic lymphocytic leukemia	Phase 2	NCT01627054	^Astex^	^125^
Imatinib	ACE2	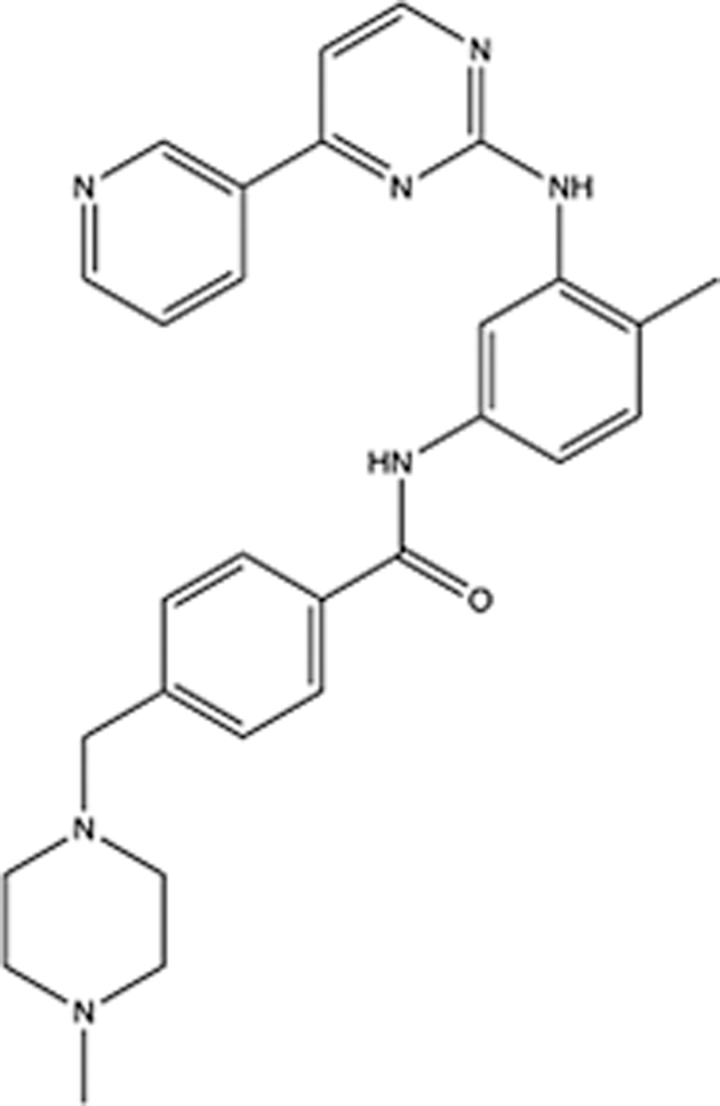	COVID-19	Phase 3	NCT04422678	^Alexandria University^	^126^
Nolatrexed	Novel thymidylate synthase	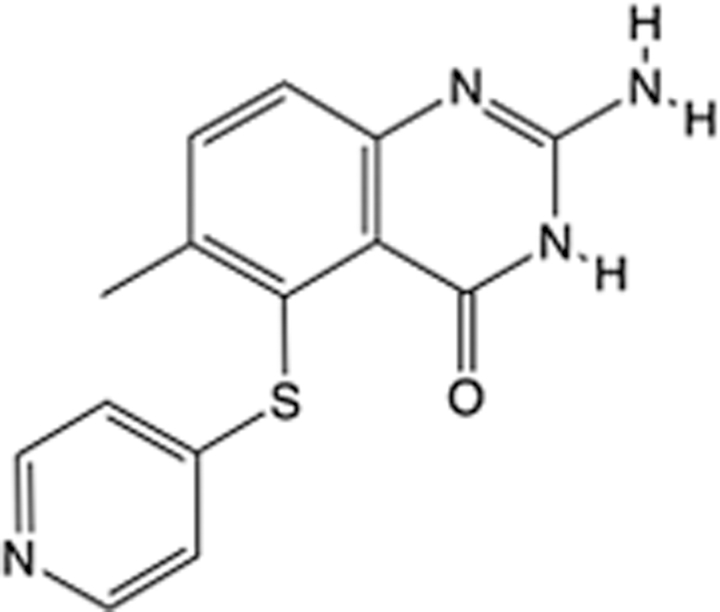	Liver cancer	Phase 3	NCT00012324	^Eximias Pharmaceutical^	^127^
LY-517717	Activated factor X	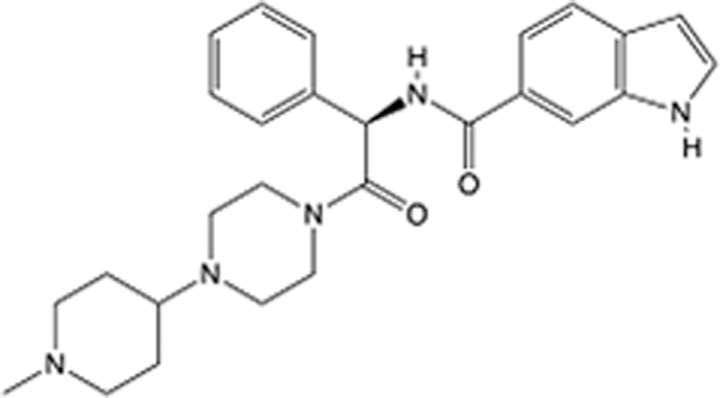	Anticoagulant	Phase 2	NCT00074828	^Lilly^	^128^
NVP-AUY922	HSP90	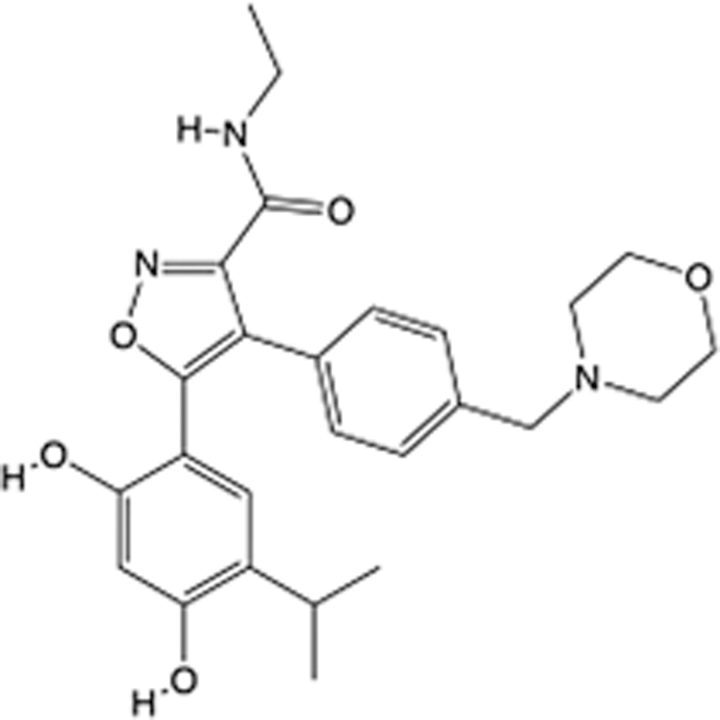	Relapsed or refractory multiple myeloma	Phase 1/2	NCT00708292	^Novartis^	^128,129^

SBDD, structure-based drug design

**Table 5:** List of clinically approved drugs discovered by LBDD.

Drug	Target	Structure	Indication	Approved in year	Clinical trial ID	Company	References
Raltegravir	HIV protease	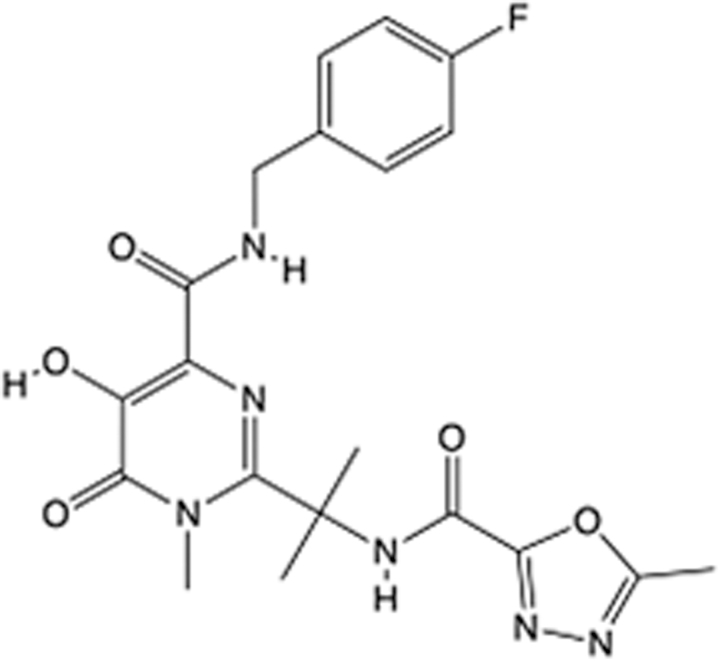	HIV infections	2007	NCT03667547	^Merck^	^130^
Nelfinavir	HIV protease	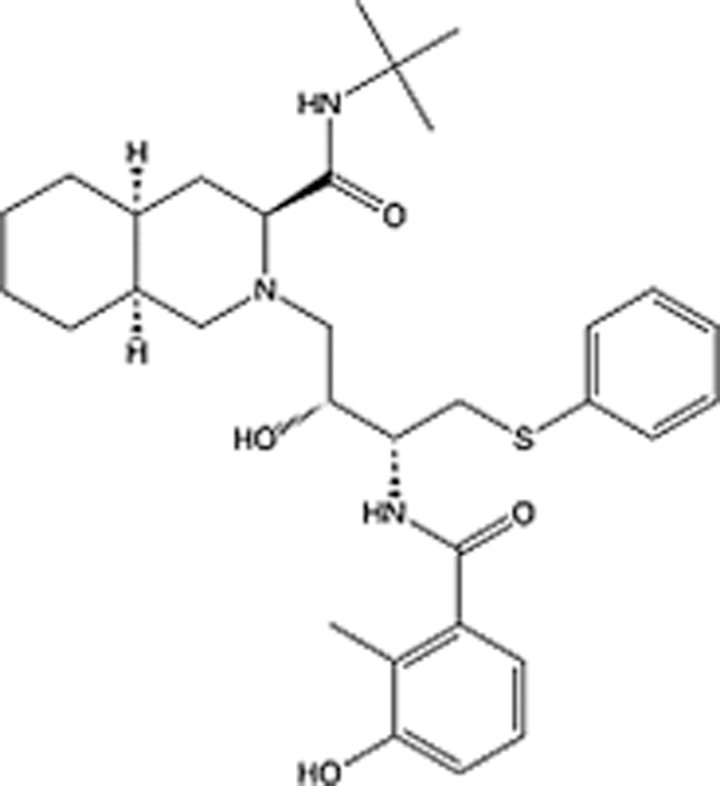	HIV infections	2000	NCT00002171	^Agouron^	^131^
Zolmitriptan	5-HT1B/1D receptor agonist	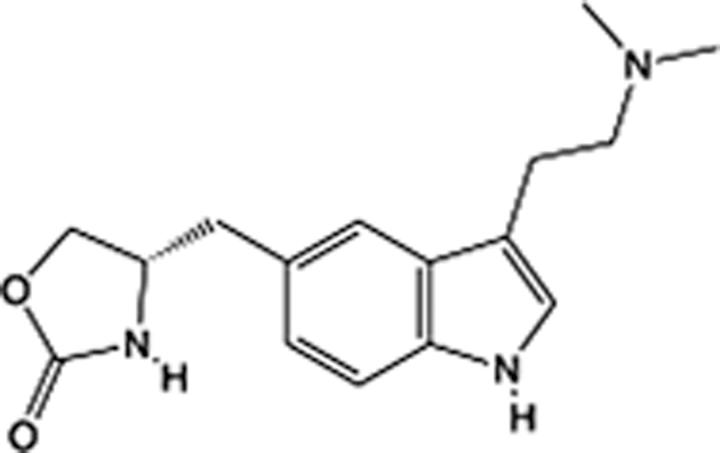	Acute migraine	1997	NCT00617747	^AstraZeneca^	^132^
Donepezil	Piperidine-class AChE inhibitor	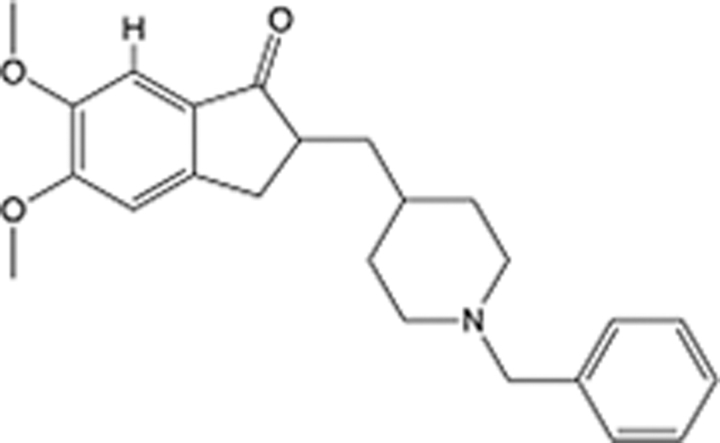	Alzheimer’s disease	1996	NCT00829374	^Eisai Inc.^	^133^
Nevirapine	Non-nucleotide reverse transcriptase	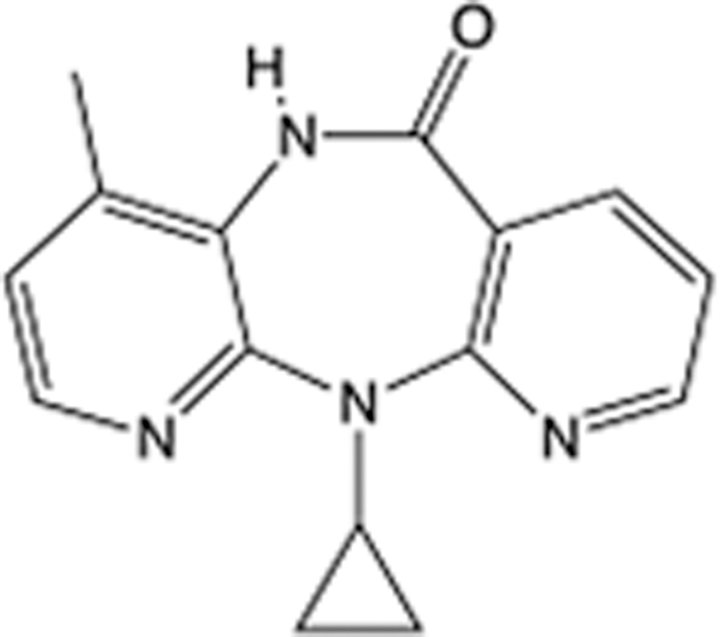	HIV infections	1996	NCT00002166	^Boehringer Ingelheim^	^134^
Indinavir	HIV protease	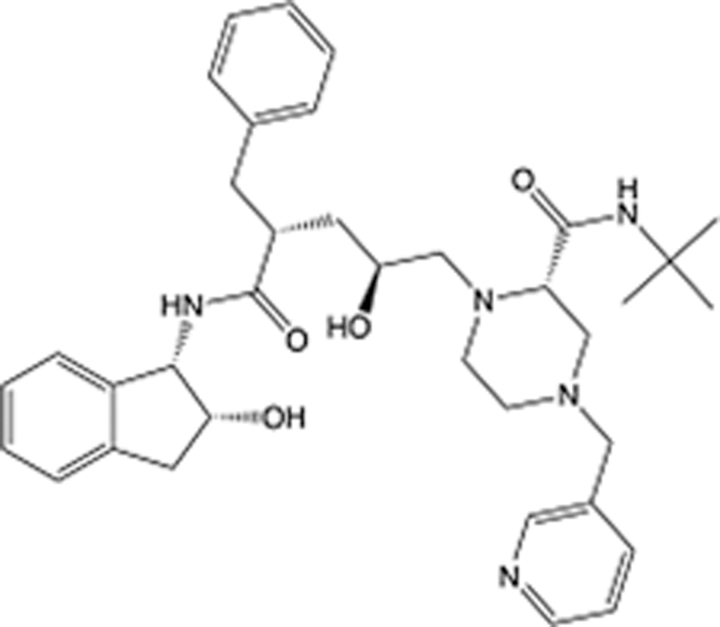	HIV infections	1996	NCT00002223	^Merck & Co^	^135^
Ritonavir	HIV Protease	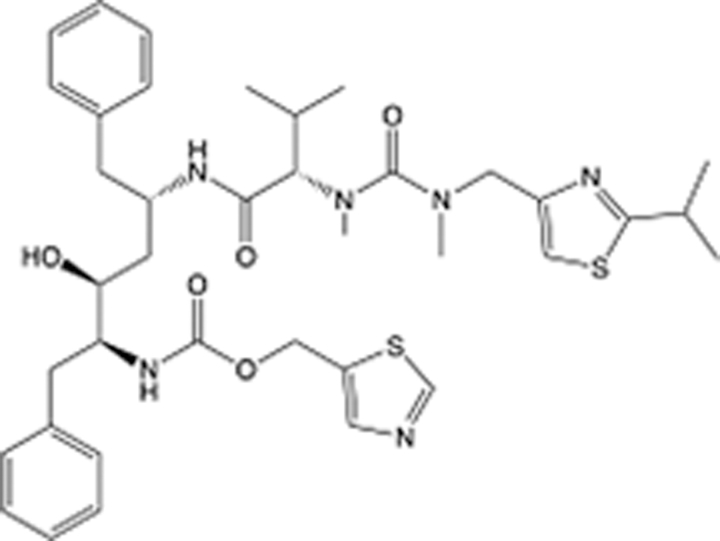	HIV infections	1996	NCT00002223	^Abbott^	^135^
Saquinavir	HIV protease	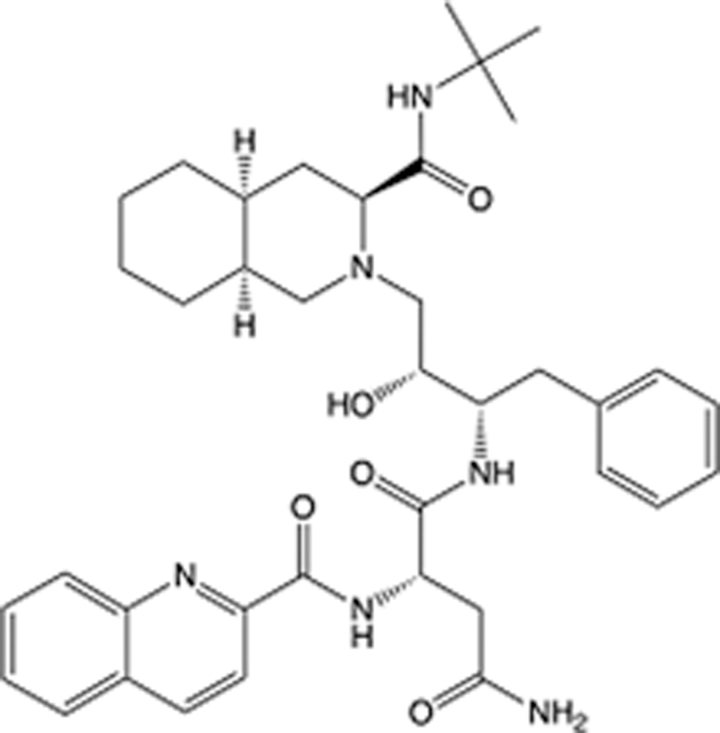	HIV infections	1995	NCT00002425	^Roche^	^136^
Losartan	Angiotensin II receptor antagonist	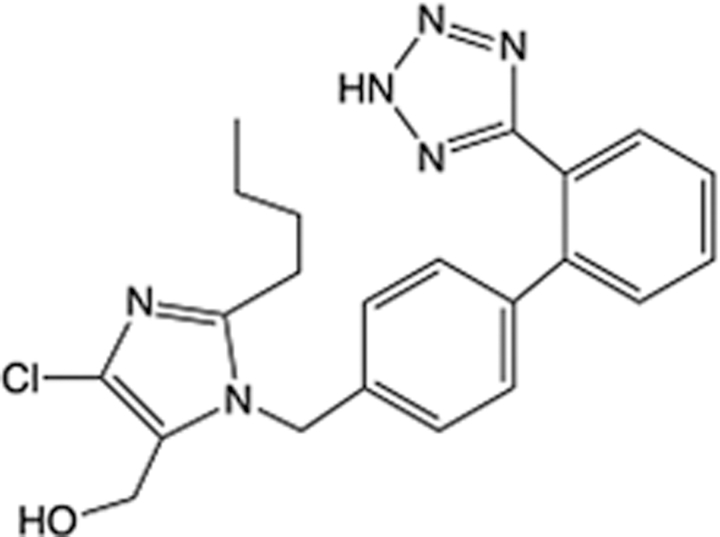	Antihypertensive	1995	NCT00617877	^Merck Sharp & Dohme LLC^	^132^
Norfloxacin	Bacterial gyrase	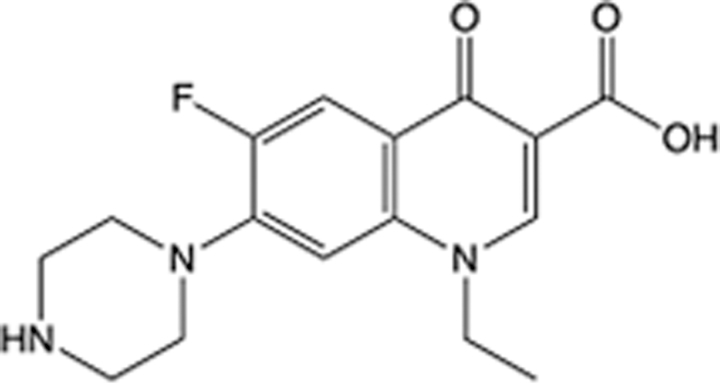	Antibacterial	1984	NCT00359853	^Hospital Clinic of Barcelona^	^137^

LBDD, ligand-based drug design.

**Table 6:** List of clinically approved drugs discovered by FBDD.

Drug	Target	Structure	Indication	Approved in year	Clinical trial ID	Company	References
Sotorasib	KRAS G12C	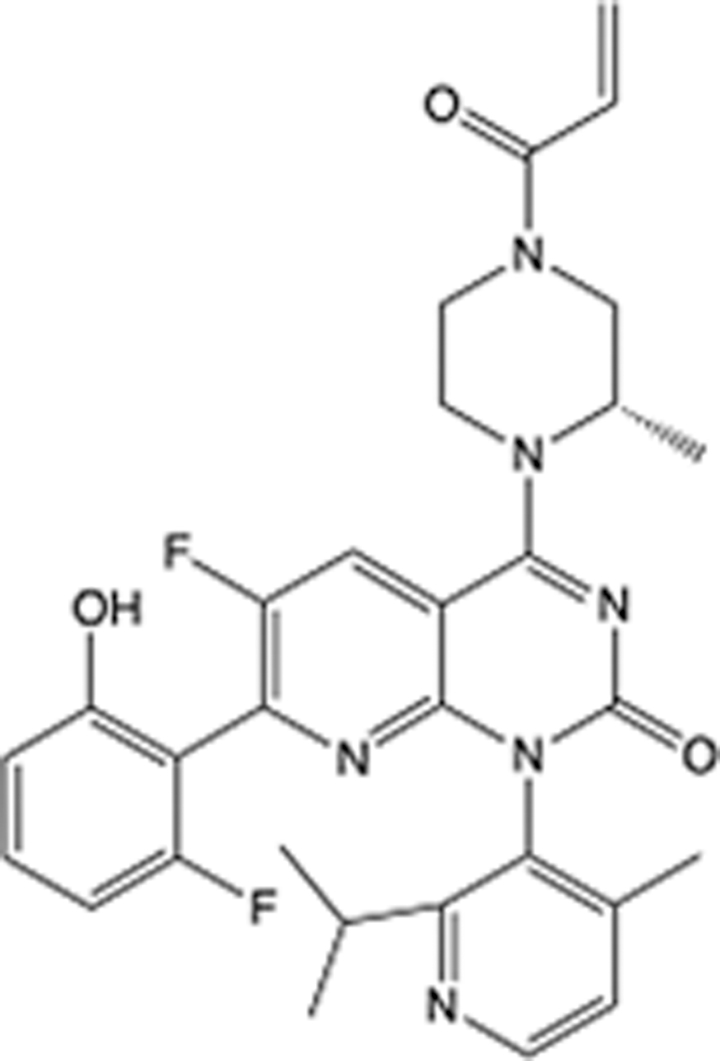	Advanced solid tumor	2021	NCT03600883	^AMGEN^	^138^
Asciminib	BCR-ABL1	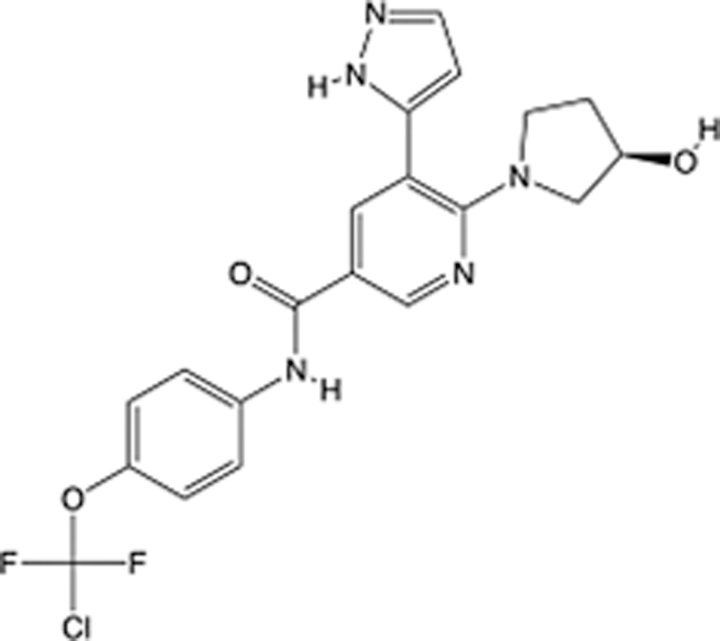	^Chronic myelocytic leukemia^	2021	NCT04666259	^Novartis^	^139^
Erdafitinib	FGFR1-4	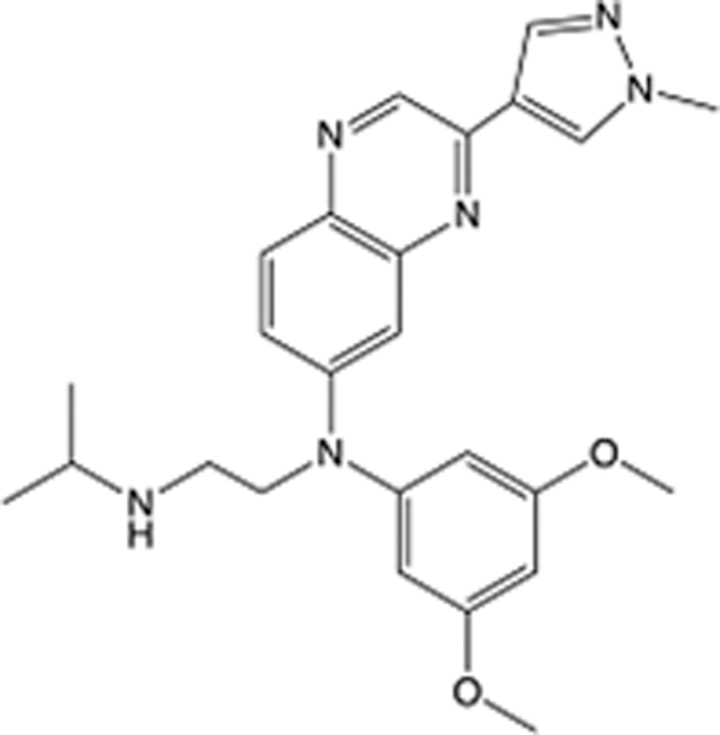	Advanced cancers and FGFR genetic alterations	2019	NCT03825484	^Astex/J&J^	^140^
Pexidartinib	CSF1R, KIT	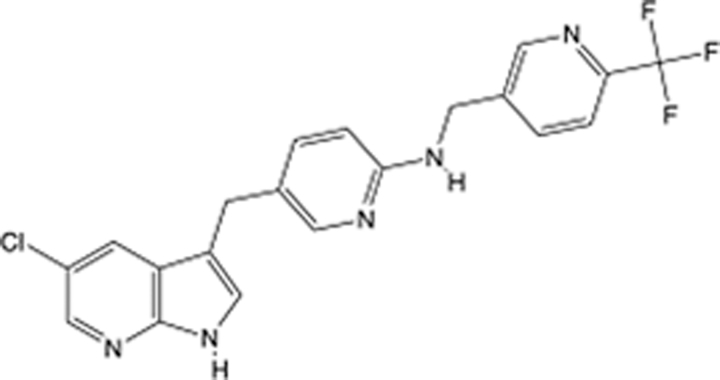	Tenosynovial giant cell tumor	2019	NCT04488822	^Plexxikon^	^141^
Venetoclax	Selective BCL-2	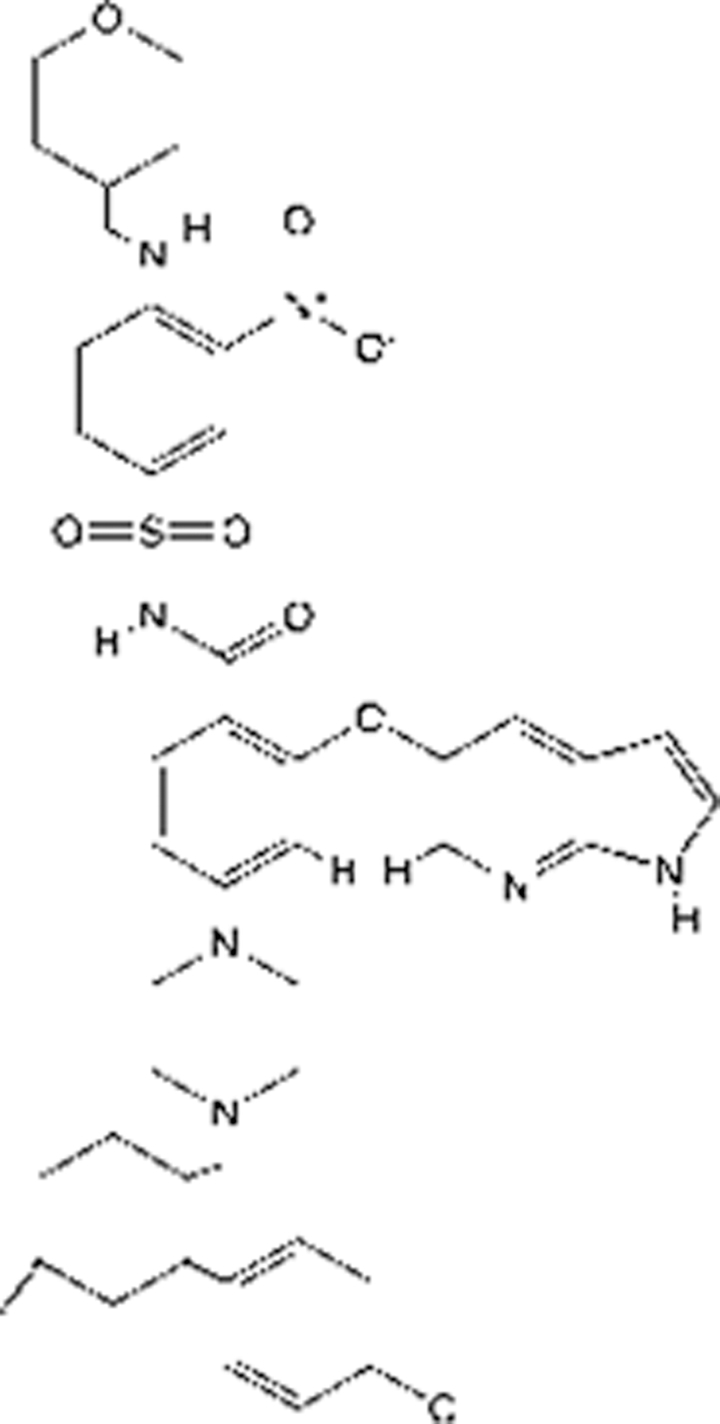	Leukemia	2016	NCT03844048	^AbbVie/Genentech^	^142^
Vemurafenib	_B-RAF_^V600E^	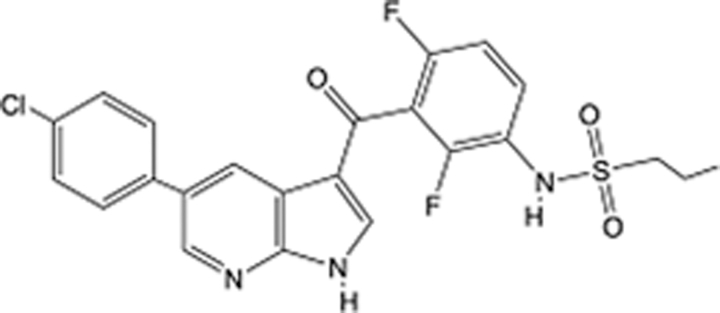	Malignancies	_2011_	NCT01739764	^Plexxikon^	^143^
Lanabecestat	BACE1	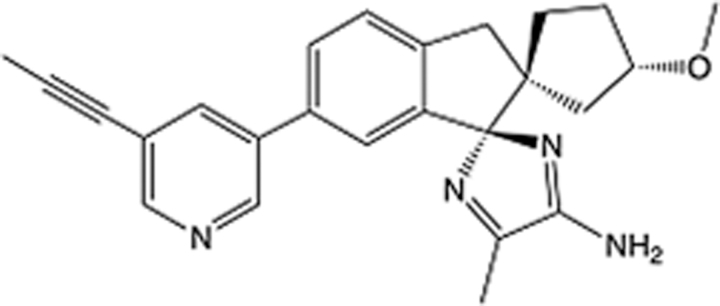	^Alzheimer’s disease^	Phase III	NCT02783573	^AstraZeneca/Lilly/Astex^	^144^
Verubecestat	BACE1/2	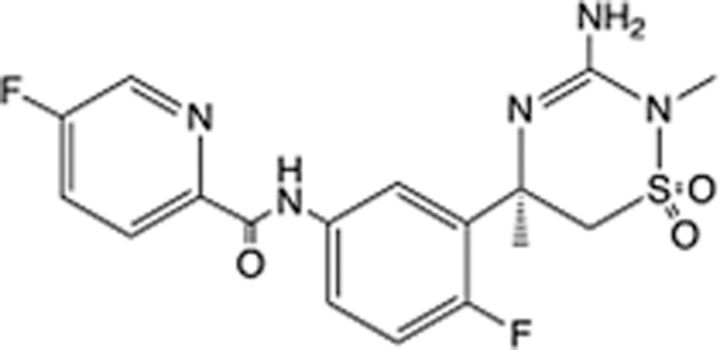	^Prodromal Alzheimer’s disease^	Phase III	NCT01739348	^Merck^	^145^
CPI-0610	BET	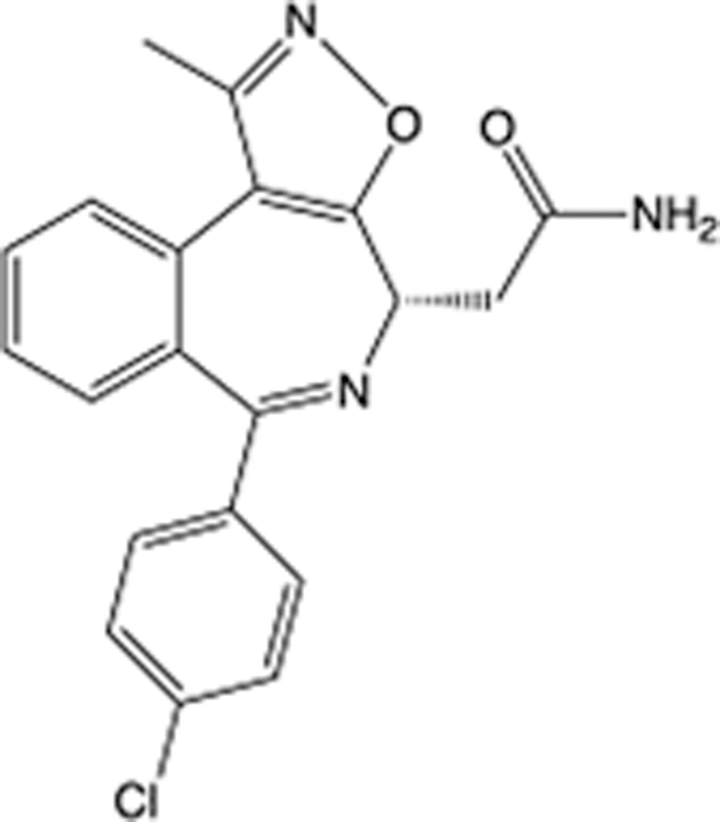	Myelofibrosis	Phase III	NCT04603495	^Constellation^	^146^
AT9283	Aurora A/B, JAK2/3	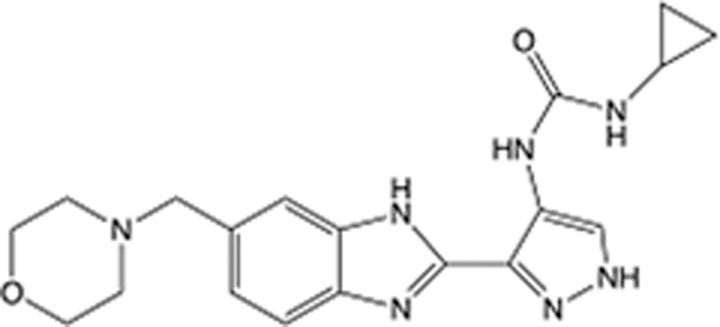	Multiple myeloma	Phase II	NCT01145989	^Astex^	^147^
Luminespib	HSP90	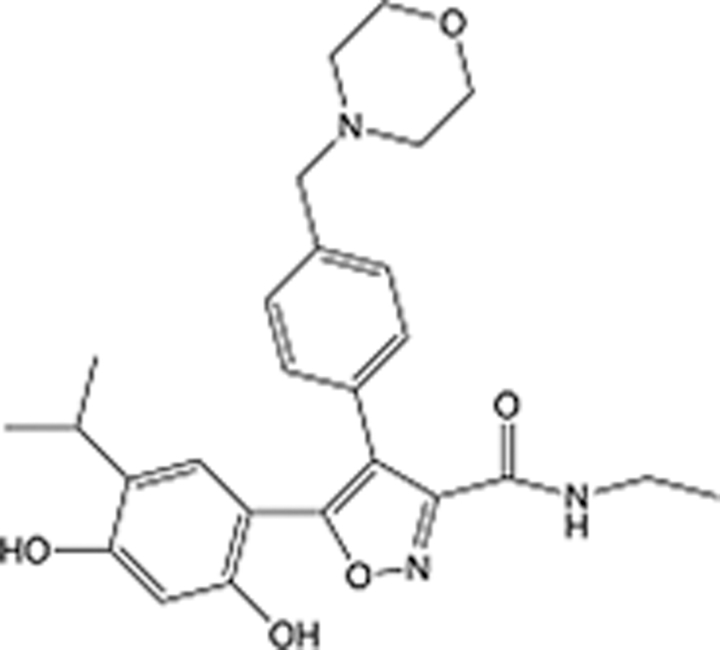	Lymphoma	Phase II	NCT01485536	^Vernalis/Novartis^	^148^
Capivasertib	pan-AKT	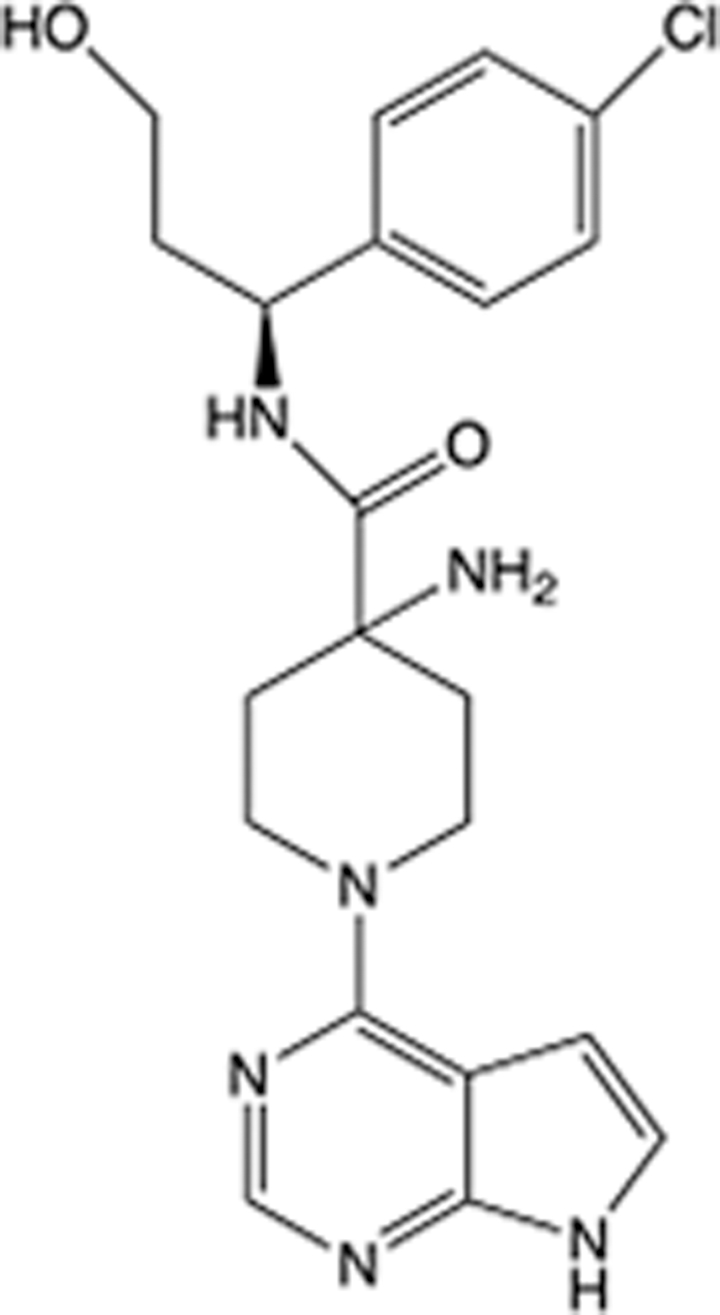	Advanced lymphoma, Advanced solid tumor	Phase II	NCT04439123	^AstraZeneca/Astex/CR-UK^	^149^
Tomivosertib	MNK1/2	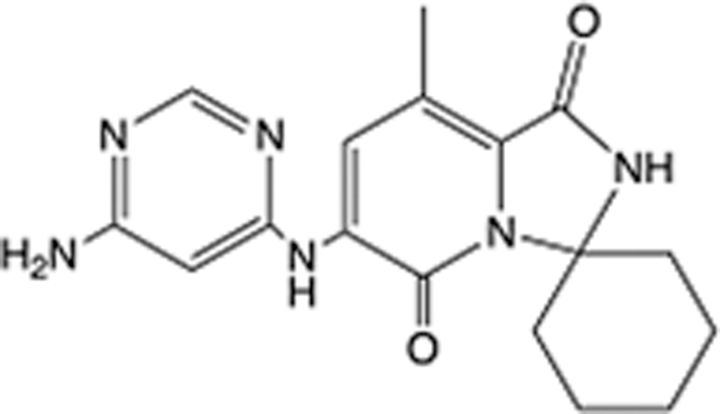	Advanced castrate-resistant prostate cancer	Phase II	NCT03690141	^eFFECTOR Therapeutics^	^150^
Zimlovisertib	IRAK4	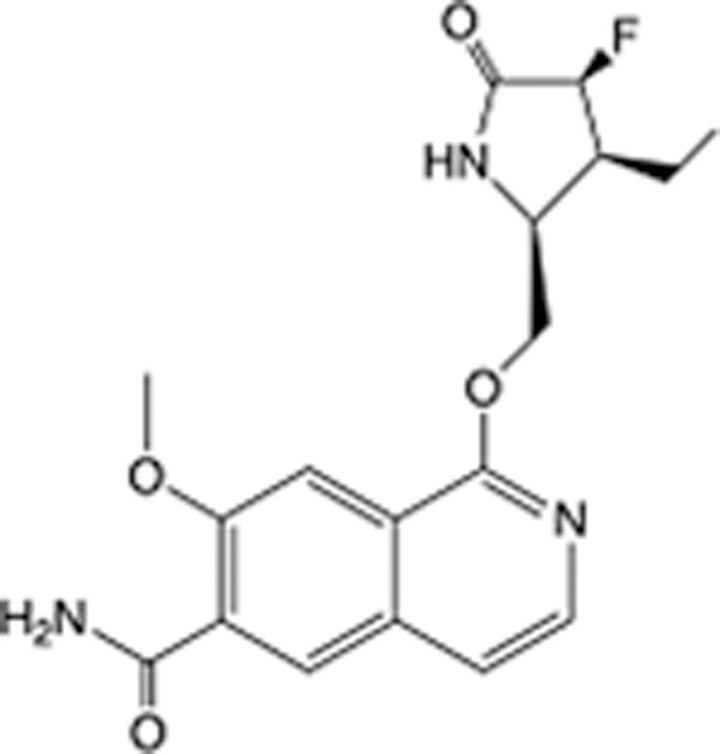	COVID-19 pneumonia	Phase II	NCT04933799	^Pfizer^	^147^
Navitoclax	Bcl-2/Bcl-xL	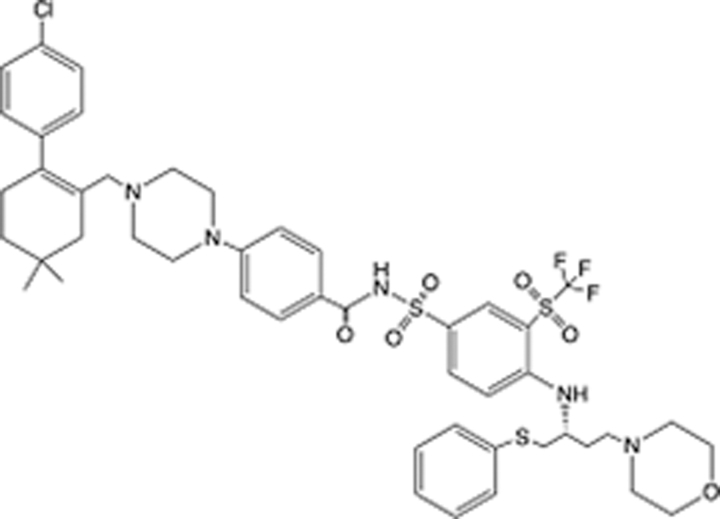	Chronic lymphocytic leukemia	Phase II	NCT00481091	^AbbVie^	^151^
GSK1070806	Soluble IL-18	N/A	Type 2 diabetes mellitus	Phase II	NCT01648153	^GlaxoSmithKline^	^152^
Tozasertib	Aurora A/B/C	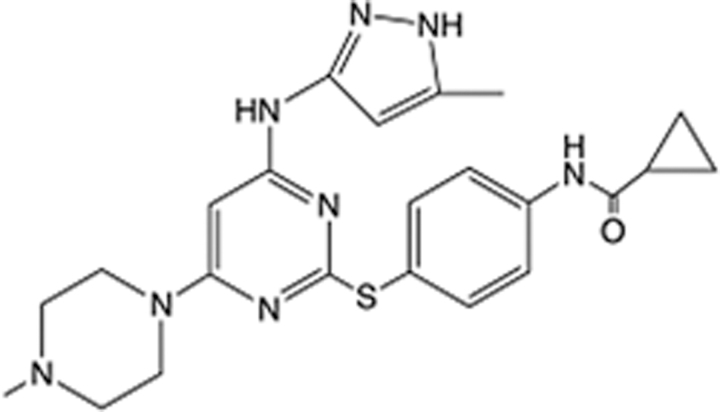	Non-small-cell lung	Phase II	NCT00290550	^Merck^	^152^
PLX51107	BRD4	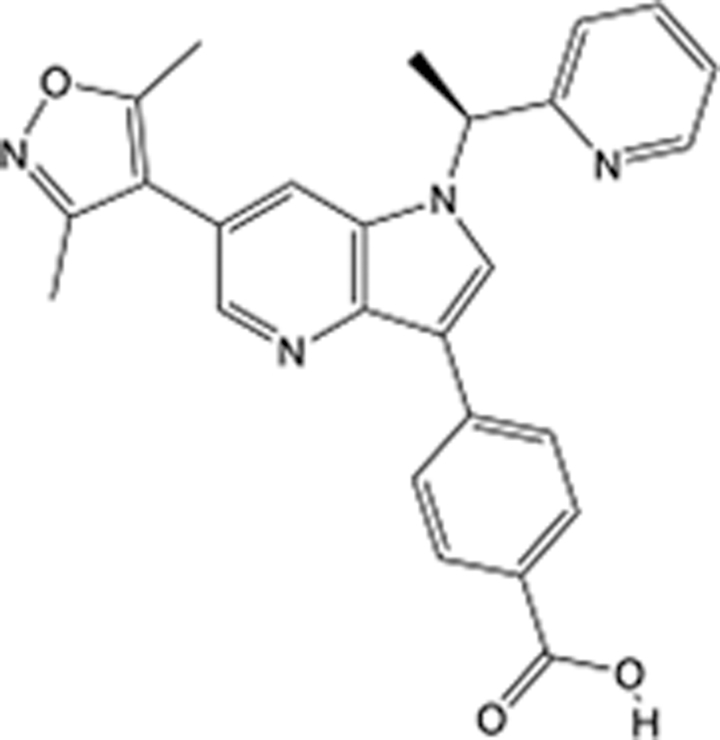	Acute graft versus host disease	Phase I/II	NCT04910152	^Plexxikon^	^153^
ASTX029	ERK1,2	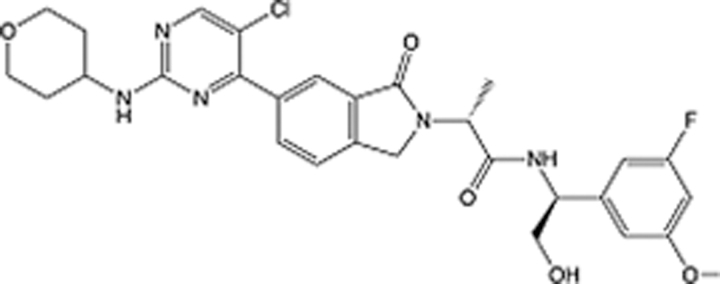	Advanced solid tumors	Phase I/II	NCT03520075	^Astex^	^154^
Tolinapant	XIAP/cIAP1	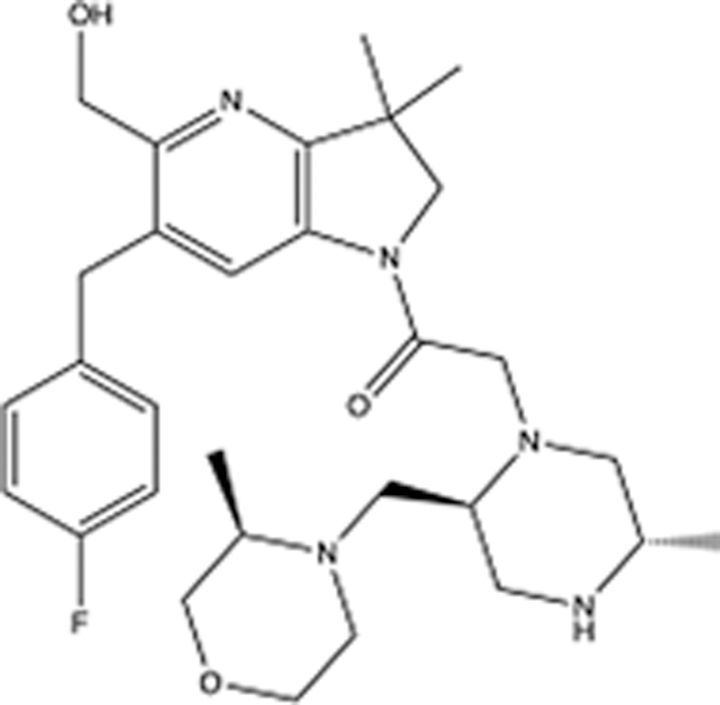	Solid tumors, lymphoma	Phase I/II	NCT02503423	^Astex^	^155^
AT13148	ROCK, AGC Kinase	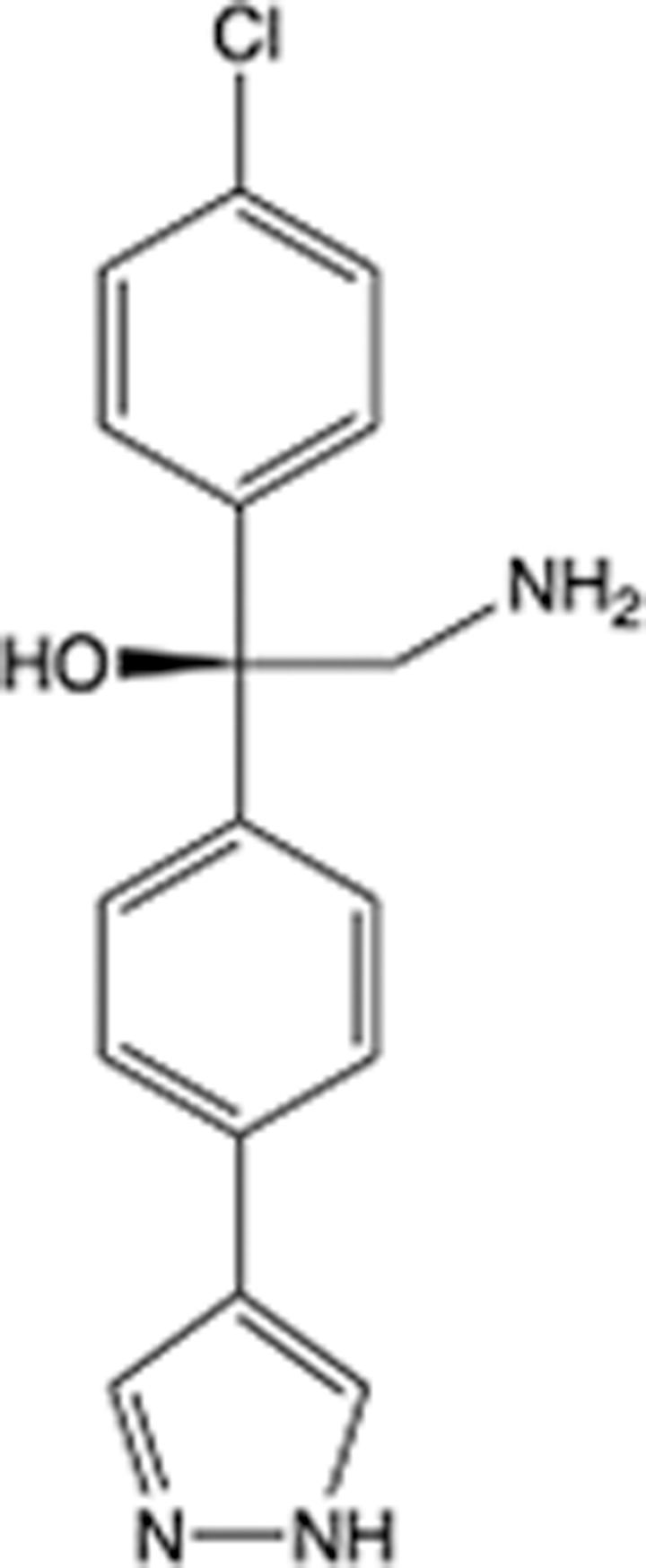	Advanced solid tumours	Phase I	NCT01585701	^Astex^	^156^
AZD3839	BACE1	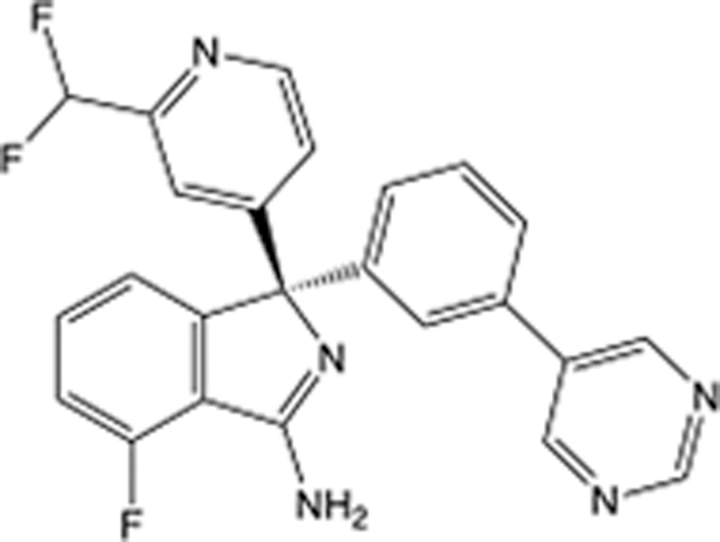	Alzheimer’s disease	Phase I	NCT01348737	^AstraZeneca^	^157^
LY2811376	BACE1	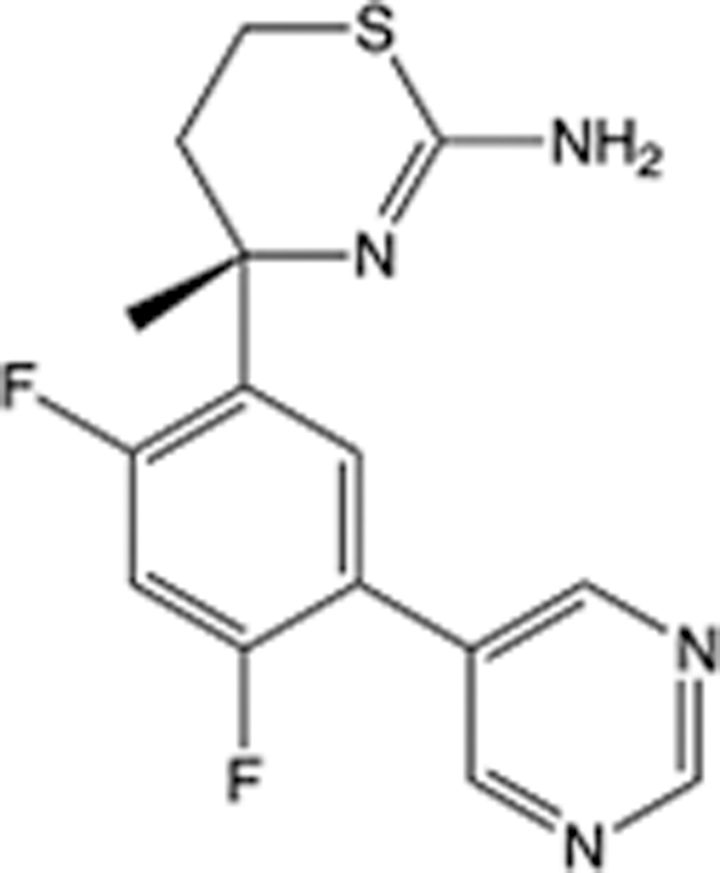	Alzheimer’s disease	Phase I	NCT00838084	^Lilly^	^158^
Mivebresib	BRD2-4	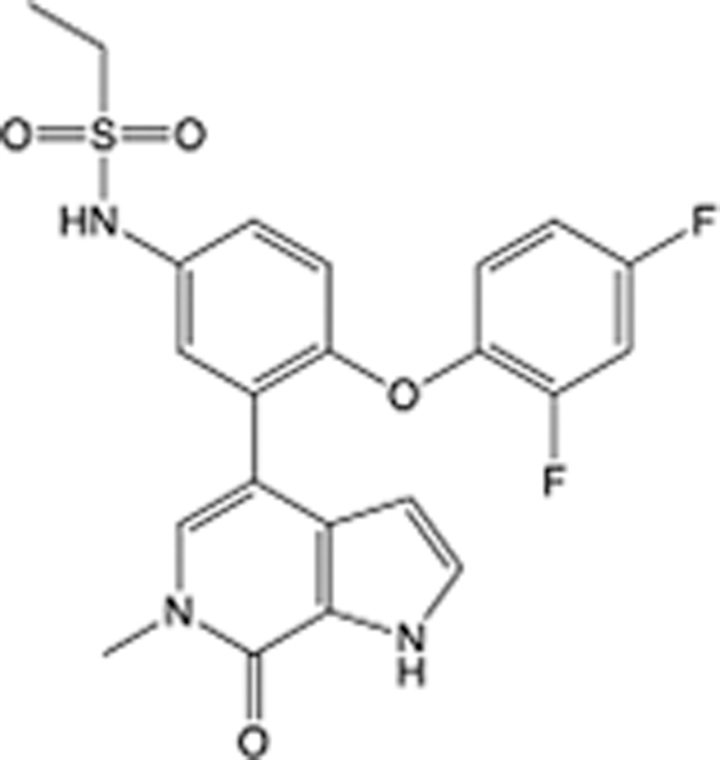	Myelofibrosis	Phase I	NCT04480086	^AbbVie^	^153^
ABBV-744	BRD2-4	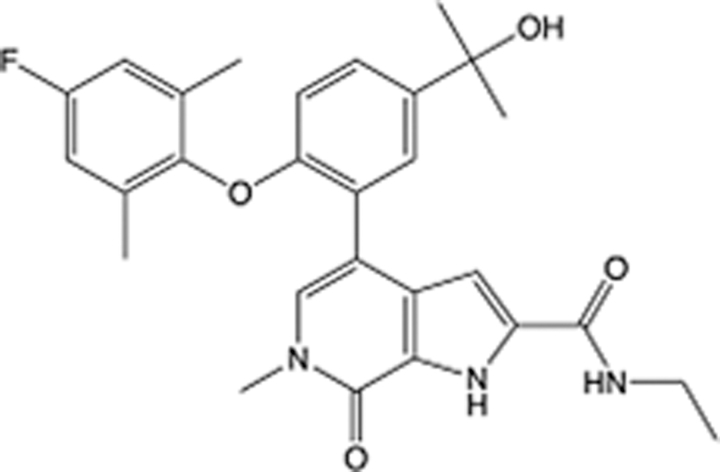	Myelofibrosis	Phase I	NCT04454658	^AbbVie^	^153^
SNS-314	Aurora B Kinase	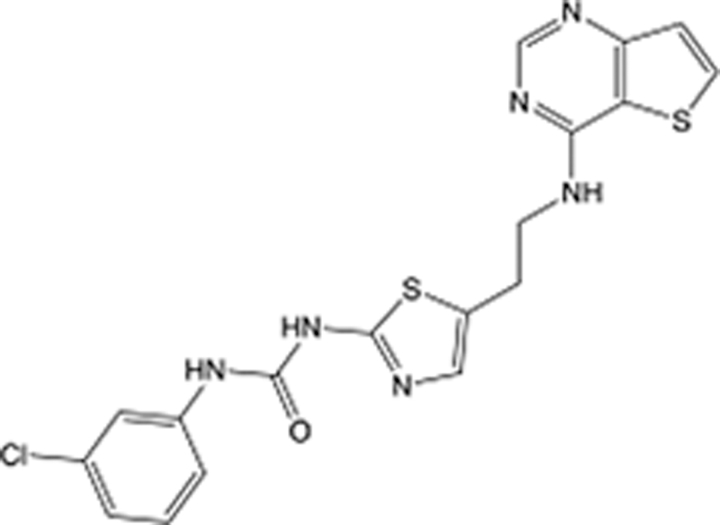	Advanced solid tumors	Phase I	NCT00519662	^Sunesis^	^152^

### Frontier topics

#### Computer-aided drug design and artificial intelligence in drug discovery

AI plays a major role in drug discovery, drug design, and the study of drug–drug interactions^159^. AI usually works by training many pretrained models, analyzing information about correlations and patterns, and then using these patterns to make predictions^160^. ML is a branch of AI and computer science that focuses on using data and algorithms to mimic human learning processes by gradually improving their accuracy. In CADD, ML can perform different tasks, such as classification, regression, grouping, and modal recognition, by learning features from many biomedical datasets^161^. ML is widely used for protein structure prediction, protein–ligand docking scoring, QSAR modeling, and pharmacokinetic prediction. As a key element in SBDD, the prediction of binding affinity prediction (BAP) of protein–ligand complexes can be efficiently achieved by a combination of structural descriptors and ML models^162^. However, the development of concise descriptors for accurate and interpretable BAP measurements remains a challenge in the field of CADD technology. Professor Debby D Wang of the University of Shanghai for Science and Technology has developed an ML-based descriptor for BAP (Intermolecular contact profiles, IMCPs), which is simple to understand, works closely with classical ML models, and provides a high level of structural information about protein–ligand complexes^162^.

With the introduction of AlphaFold2, a tool designed for protein structure prediction, the rapid development phase of AI-driven drug design has been ushered in. The neural network of AlphaFold2 can predict the structure of a typical protein within minutes, achieving laboratory-level accuracy^163^. This breakthrough significantly propels advancements in biology and drug development. The protein structure prediction model constructed by AlphaFold2 introduces a new paradigm for small-molecule drug design in SBDD, offering different levels of confidence and accuracy^164^. Furthermore, the research team of Professor Mingyue Zheng at the Shanghai Institute of Pharmaceutical Sciences, Chinese Academy of Sciences, proposed an AI method, pairwise binding comparison network (PBCNet), for lead compound optimization. The method adopts a twin graph convolutional neural network architecture to predict the relative binding affinity between a set of similar ligands by comparing the differences in their binding patterns, which can strike a better balance between computational speed and accuracy. Additionally, the research team designed TransformerCPI2.0, which is a tool that can generalize and learn across proteins and compounds to identify new targets for existing drugs based on the reverse application of the concept^165,166^. This series of research results highlight our continuous innovation and progress in the field of AI-CADD. In summary, the introduction of these new methods provides a more efficient and precise tool for drug design, laying a solid foundation for future drug development and innovation.

#### The role of CADD in medicine

*The role of CADD in Alzheimer’s disease*: Alzheimer’s disease (AD) is the most common form of dementia in the elderly, with a high prevalence of cognitive decline and non-cognitive neuropsychiatric symptoms, severely affecting patients’ daily life and behavioral abilities^167^. Currently, CADD technology has been widely used in AD diseases and other neurodegenerative diseases, mainly for structure or ligand drug design with AChE, BACE1, mAChR, nAChR, Tau protein, and other targets^168^. Manoharan and Ghoshal^169^ developed a ligand- and structure-guided virtual fragment screening method that combines the shape, electrostatic and pharmacodynamic features of known fragment molecules with protein-conjugated crystal structures with the aim of identifying chemically and energetically viable small fragment ligands that bind to the active site of BACE1, which can be used to identify lead molecules against BACE1. Additionally, Domínguez *et al*.^170^ proposed a computer-assisted scheme based on pharmacology and a set of interaction requirements with the target, combined with molecular docking and MD simulations, to screen a set of carbazole-containing compounds with substituted aniline fragments, which showed an increase in affinity for BACE1 by more than three orders of magnitude, as well as an inhibitory effect on amyloid-beta (Aβ) aggregation and affinity for AChE. Similarly, Job *et al*.^171^ designed seven molecules that may act as dual inhibitors of Aβ and p-tau (L1-7), and the binding affinities of these molecules for Aβ and p-tau were confirmed by molecular docking analyses, and the highest similarity to ApoE was found for L3 by MD simulation data, and these have the ability to cross the blood–brain barrier. Moreover, CADD technology has been used for the optimization of existing inhibitors. Meine *et al*. optimized the DYRK1A inhibitor (KuFal194); the 7-chloro-1H-indole-3-carbonitrile from KuFal194 was used as a fragment template to develop a less lipophilic DYRK1A inhibitor. And a series of indole-3-carbonitrile was also designed and evaluated as potential DYRK1A ligands by molecular docking studies^172^. Furthermore, in addition to CADD technology, technologies such as ML and AI are also widely used in AD diseases^173^, and the integration of these technologies is highly likely to overcome the world’s medical problems.

*The role of CADD in COVID-19 therapeutics*: In accordance with the drug repurposing strategy, researchers repurposed carvedilol, a nonselective beta-adrenergic blocker originally designed to treat hypertension, for use against severe acute respiratory syndrome coronavirus 2 (SARS-CoV-2) infection using the CADD technique (in silico molecular docking and MD simulation) and identified the molecular mechanism and potential molecular targets of carvedilol’s anti-SARS-CoV-2 resistance by evaluating the interactions of carvedilol with viral proteins^174^.

Moreover, using a molecular operating environment (MOE), structure-based virtual screening (SBVS) was carried out with SARS-CoV-2 proteins. The University of California San Francisco and the University of Oxford have embarked on a collaborative study based on structure optimization, virtual screening and primary screening with X-ray crystallography to identify large structural domain binding compounds from computational designs and provide structural information to guide compound optimization^175^. The authors found that the determination of 150 Mac1-ligand crystal structures supported the discovery and optimization of 19 low- and subμM compounds that fall into eight different scaffolds and chemotypes. The compounds and structures will contribute to the development of antiviral drugs targeting the NSP3 macrodomain of SARS-CoV-2^175^. Furthermore, Professor Haitao Yang’s research team at ShanghaiTech University identified a mechanism-based inhibitor (N3) by means of CADD technology and subsequently determined the crystal structure of the SARS-CoV-2 (M^pro^) protein in a complex that binds to this compound. More than 10 000 compounds were also tested as inhibitors of M^pro^ by a combination of structure-based virtual screening and high-throughput screening. Six of these compounds inhibited the M^pro^ protein, with IC50 values ranging from 0.67 to 21.4 μM^24^.

In addition, the research team integrated five databases, namely, the ethnobotanical database, IMPPAT, PhytoHub, AromaDb, and Zinc, to compile a set of 7809 natural compounds. The researchers used a rigorous computational methodology to identify lead molecules from the screened set of compounds through docking, dynamic simulation, combined free energy and density functional theory (DFT) calculations and confirmed molecules with better inhibitory effects from theaflavin and ginkgetin against SARS-CoV-2 and Omicron variants^176^. Based on the virtual screening stage, Hamed *et al*.^177^ adopted a structure-based drug design approach, that is, molecular docking by MOE software and AutoDock Vina software and MD simulation analysis, and found that cyanidin-3-rhamnoglucoside, the active ingredient in *Ficus carica*, binds well to the M^pro^ protein and the ACE2 protein, with a free binding energy of approximately −200 kJ/mol, suggesting that the fruit of *F. carica* is useful as an adjunctive therapy for the treatment of symptoms associated with COVID-19. The results of this study demonstrated the effectiveness of the CADD screening strategy for the rapid discovery of drug leads with clinical potential for novel infectious diseases for which no specific drug or vaccine is available.

*The role of CADD in cardiovascular diseases*: Cardiovascular diseases (CVD) are considered the first cause of death globally^178^. It is common to have significant coronary heart disease, myocardial ischemia and hypertension, as well as diabetes-related complications^179^. Early scientists used CADD technology applied to cardiovascular disease research and successfully discovered the ACE1 inhibitor captopril^124^. With the development of genomics and molecular genetics, more and more effective targets have been identified. G protein-coupled receptor (GPCR) kinases (GRKs) are mainly involved in the desensitization of GPCRs. The key role of GRK2 in diseases such as cardiac hypertrophy, hypertension, and heart failure has facilitated the study of pharmacological inhibitors of its activity. Several studies have shown the use of the crystal structure of GRK2 to perform a docking-based virtual screen for potential inhibitors of the interaction of GRK2 with the Gαq protein^180^. As a result, GRK2 inhibitors capable of reducing histamine H1 receptor desensitization and GRK2 translocation to the plasma membrane mediated by the structural domain of the GRK2 homologous G protein signal transduction regulator were identified^180^. Additionally, stromal cell-derived factor-1α (SDF-1α, CXCL12) mediates the migration of circulating cells to desired sites and promotes tissue development. Its protective role after myocardial infarction. Several studies have shown SDF-1α analogue peptides designed by SBDD and screened four analogue peptides. One of these analogue peptides, SDP-4, promoted cardiac regeneration in myocardial infarction animals more than native SDF-1α. The results confirm that the SDP-4 peptide is an excellent candidate for cardiac regeneration in acute myocardial infarction^181^.

*The role of CADD in cancer*: Identifying potent drug inhibitors for key sites of core targets remains a challenge. Protein–protein interactions between the structural domains of the BCL6 protein and inhibitory factors have emerged as the most promising idea for anticancer drug development. Chen *et al*.’s^69^ team screened a library of small-molecule compounds for similar fragments based on NMR techniques and used CADD and X-ray crystallography to discover a key inhibitor (15f) that was more than 100-fold more potent against the BCL6 structural domain. Similarly, fibroblast growth factor receptor (FGFR) kinase is a promising method for structure-activity screening of compounds. Wu *et al*.^182^ developed a ML based on FGFR-specific scoring function RTKscore and subsequently synthesized the bioactivities of 66 pyrazolo[3,4-d]pyridazinone derivatives based on the SBDD technique and evaluated their activities against FGFR using kinase inhibition, lung cancer cell proliferation assays to explore the compound structure–activity relationships. Meanwhile, studies have shown that a series of indazole derivatives have been designed and synthesized using CADD strategies, and their inhibitory and anti-hepatocellular carcinoma activities against FGFR4 kinase have been evaluated^183^. Furthermore, several studies have shown the design and synthesis of a new novel nitrogen-containing heterocyclic compound based on CADD technology, which can inhibit the proliferation and invasion of U251 glioma and MCF-7 breast cancer cells by regulating tumor-associated genes and induce apoptosis and cell cycle arrest^184^. In addition, Ma *et al*. designed and synthesized a series of anilide (dicarboxylic acid) shikonin esters targeting the PI3K/Akt/mTOR signaling pathway and evaluated their anti-breast cancer effects. Then through three rounds of screening using CADD methodology, they initially obtained 16 novel aniline (dicarboxylic acid) shikonin esters and identified them as excellent compounds^185^.

*The role of CADD in HIV*: Pathogenic viral infections are globally prevalent and threaten human health. Some viruses, such as HIV, hepatitis B virus (HBV), SARS-CoV-2 Omicron, and monkeypox virus, cause persistent infections that are difficult to cure and may develop into a variety of other diseases^186–188^. Several studies have shown that the design of non-peptide HIV protease inhibitors based on the cyclooctylpyranone lead structure with substitutions at the meta-position of the aryl ring has yielded compounds such as the carboxyamide derivatives 36,36 which have a markedly increased binding affinity for the HIV-1 protease complex, which is most likely due to additional interactions between the amide substituent and the S3 region of the protease^189^. Parvez *et al*.^190^ developed various ML-based predictive models for identifying highly active compounds for anti-HIV integrase. The validated models were further used for the virtual screening of potential compounds in the ChemBridge library. Among the six highly active compounds predicted by the selected models, compounds 9103124, 6642917, and 9082952 showed the most reasonable binding affinity and interaction stability with HIV integrase active site residues Asp64, Glu152, and Asn155^190^. Additionally, Annan *et al*.^191^ used a CADD approach to propose novel analogues of efavirenz as potential direct inhibitors of HIV reverse transcriptase. Furthermore, with the development of CADD technologies, ML and deep learning have been successfully used to facilitate rapid innovation in the virtual screening of anti-HIV drug candidates^192^.

*The role of CADD in natural products*: As an important part of the world’s medicine, natural products and TCM have a wide influence in the global arena. Its richness in small molecular compounds is a valuable resource for the design and screening of new drugs. However, due to the difficulty in estimating the number of natural molecule compounds and the problems associated with drug discovery (e.g. ADMET properties and pharmacokinetic evaluation), it is difficult for researchers to optimize or screen applicable compounds through traditional structure modification. This may be a new development trend in the future. Several studies have shown that the best 3D QSAR pharmacodynamic model Hypo1 was generated based on a training set of flavonoids using the HypoGen programme in Discovery Studio 2016^193^. Hypo1 model was also used to perform a virtual screening from the TCM and MiniMaybridge databases. And 10 AChE inhibitors were obtained by molecular docking analysis. Interestingly, de Novo Evolution was designed for these 10 derivatives and finally three most promising AChE candidate inhibitors were obtained^193^. In addition, Chen *et al*.^194^ identified cynarine from the TCM database as a squalene synthase inhibitor candidate compound based on pharmacodynamic properties, molecular docking studies and MD simulations. Similarly, Chinnasamy *et al*. performed virtual screening and molecular docking studies using the TCM database to screen scutellarin (TCM3290) and calculated by DFT calculation at B3LYP/6-31G^**++^ level to explore the stereo-electronic features of the molecule. The results demonstrated that the binding mode of scutellarin (TCM3290) inhibitor was more stable within the binding site of phospholipase A2 (PLA2) by 100 ns of MD simulations^195^. Meanwhile, in order to elucidate the mechanism of action of Chinese herbal medicines for the treatment of COVID-19, Dai *et al*.^196^ innovatively used HTS2 technology in combination with bioinformatics methods and CADD to evaluate 578 Chinese herbal medicines and 338 reported anti-COVID-19 Chinese herbal formulas, and ultimately identified the core targets and the potential active ingredients in these herbal medicines.

In addition, an integrated CADD and AI system has also been widely applied in predicting active components in natural products. Studies have used deep learning combined with CADD to predict the active components of Xiao Shuan Tong Luo prescriptions related to stroke, accelerating the discovery of drugs for stroke prevention and treatment^197^. However, the safety of TCM also raises concerns. ML can continuously optimize models by choosing appropriate algorithms. Chen *et al*.^198^, for example, constructed a model using ML to screen for hepatotoxic components among many compounds in TCM.

*Combining nanomedicine and CADD technology*: Nanomedicine is an important medical subfield, and nanomedicines have shown great potential for improving therapeutic efficacy and reducing the side effects of conventional drugs. Several studies have focused on redesigning the ligand and applying it across the blood–brain barrier to mutant brainstem glioma sequences using CADD tools and designing a set of vitamins with nanomedicines that interact with brainstem glioma proteins^199^.

Targeted drug therapy is limited by a narrow therapeutic window, short drug half-life and high risk of bleeding complications. However, nanomedicine-based peptide therapy can protect protein-based drugs from enzymatic degradation, improve therapeutic efficacy, and reduce adverse reactions in clinical treatment^200^. The physicochemical properties of peptide drugs can be improved by drug design, and the rational design of peptide therapeutics can solve the problems of short plasma half-life and low bioavailability. Furthermore, the selective incorporation of D-amino acids, disulfide bonds, and cyclization was found to alter the peptide backbone and chemistry and improve peptide stability based on CADD technology^201^. In addition, the formation mechanisms of 1,4-dihydropyridine drug nanosuspensions, such as stable intermolecular hydrogen bonding, adsorption, and hydrophobicity, have been confirmed by experimental validation and computer-aided drug design^202^.

#### Molecular dynamics in drug discovery

Yasir *et al*.^203^ used CADD technology to design novel inhibitors against γ-aminobutyric acid aminotransferase (GABA-AT) based on 530 000 compounds from the Korean Chemical Bank (KCB) for pharmacodynamic analyses; biological analyses of the initial 50 compounds; and discovery of four potent inhibitors based on molecular docking, MD simulation, and free energy calculations. In addition, as the conformation of proteins is dynamic, small conformational changes involving the movement of residue side chains can affect the binding between ligands and protein targets; moreover, analyzing the dynamic structure of proteins can be useful in the design of drug molecules^204^. MD simulation is a key technique for studying the dynamic behavior of proteins and is based on Newton’s equations of motion; this method can be used to predict the trajectory of each atom in a molecular system over time. The main applications of MD simulation in CADD include prediction of binding conformations, binding free energies, binding sites, and protein kinetic and functional analyses. The crystal-resolved structure of HIV integrase is considered to be incapable of receptor structure-based drug design due to the absence of drug-binding sites. Howard Hughes Medical Institute researchers performed an MD simulation of HIV integrase, discovered a hidden binding site in the crystal-resolved structure, and demonstrated in subsequent X-ray diffraction experiments that known inhibitors indeed bind to this new binding site^205^. Moreover, Merck’s drug development based on a new HIV binding site led to the discovery of the drug L870810 (CAS No: 410544-95-5), which has potent antiviral activity^206^. Ahmed *et al*.^207^ further demonstrated that MD simulation could be a more powerful tool for studying the dynamic interactions between potential small-molecule drugs and their target proteins.

#### Drug resistance

Drug resistance represents a significant challenge in the clinical treatment of diseases. CADD can be used to predict the interactions between a drug and multiple targets, enhancing its binding affinity with mutant proteins that may confer resistance. Through techniques such as molecular docking and energy evaluation, researchers can design drug molecules with improved resistance profiles. Studies have designed a compound named ETX0462, which has demonstrated potent in-vitro and in-vivo activity against *Pseudomonas aeruginosa* and gram-negative ESKAPE pathogens, suggesting the potential for the clinical development of novel antibiotics against drug-resistant strains^208^. A recent study by Pasala *et al*.^209^ employed CADD to predict potential new targets in *Helicobacter pylori* strains.

Furthermore, the SBDD method allows for the continuous improvement of drugs based on the structures of targets associated with drug resistance. Even for FDA-approved drugs targeting known proteins, there is often a need for further SBDD work in the clinical setting. For example, some drugs might need to be redesigned to optimize their selectivity more effectively. One such case involves erdafitinib, which, after targeted design modifications, exhibited increased selectivity toward fibroblast growth factor receptors compared to the original drug molecule^210^. Therefore, the ongoing development and refinement of CADD techniques offer valuable tools in the battle against drug resistance, which allows for the design of more effective and selective therapeutic agents against a wide range of diseases. These efforts are crucial in addressing the growing global challenge of drug resistance in various pathogenic organisms and provide hope for more effective treatments in the future.

#### CADD dynamics of international cooperation

The global CADD market has had a positive impact throughout the COVID-19 pandemic. Computational technologies, especially computer-assisted virtual screening and molecular modeling, can play a key role in understanding the structural aspects of multiple molecular targets of coronaviruses as well as identifying new lead molecules. In addition, research institutes and biocompanies are working to combat the global pandemic. Additionally, in collaboration with several research institutions, the Chinese Academy of Sciences has developed a platform for target prediction and virtual screening for the discovery of anti-COVID-19 drugs based on CADD technology (D3AI-CoV) (http://www.d3pharma.com/D3Targets-2019-nCoV/D3AI-CoV/index.php), which can be used as a rapid online tool for predicting potential targets for active compounds and for the identification of active molecules against COVID-19-specific target proteins^211^.

Collaboration with academic institutions will remain key to expanding the CADD toolbox and exploring new approaches. Boehringer Ingelheim (BI) developed the Computational Chemistry Framework (CCFW) as a central hub that can connect front-end applications used by medicinal chemists (e.g. Marvin, Spotfire, D360) with back-end computational chemistry calculation engines^212^. This approach facilitates predictive modeling and workflow exchange between international collaborative teams. Moreover, the DataPype platform, which is a one-stop computer-aided drug design automation platform that was codeveloped by the Trinity Biomedical Sciences Institute in Ireland and Integral University in India, accelerates the virtual screening process for new compounds^213^. Furthermore, the US-based Mydecine Innovations Group, Inc., announced the official launch of its computer-simulated drug discovery program in collaboration with a team of researchers from the University of Alberta. The program focuses on designing AI and ML-based drug screening and development methods. These collaborations may present extremely attractive opportunities for investors in the global CADD sector.

### Challenges

#### Analysis of biological target structures

CADD research necessitates access to reliable high-resolution structures of biological targets. While X-ray crystallography can be used to study some small and well-ordered biological molecules, most known target proteins, such as transmembrane receptors, are either difficult to crystallize or do not meet the required conditions for crystallization. This limitation prevents high-resolution structural analysis of these target proteins using crystallographic methods, hindering the application of CADD technology. As a result, it is essential to employ a combination of various techniques, such as X-ray crystallography and cryo-electron microscopy, to thoroughly explore the structures of drug targets^214^.

#### Algorithm accuracy

Additionally, the algorithms and models utilized in CADD must offer accurate predictions and effective optimization strategies to guide drug discovery and design. However, existing algorithms still have room for improvement to enhance their accuracy. Currently, it is difficult to identify active compounds with novel skeletons with similarity searches based on 2D/3D fingerprints. Moreover, the accuracy of molecular docking, which involves small molecular compounds and protein structures, is not sufficiently high.. Furthermore, it is difficult to accurately evaluate the binding strength between proteins and ligands with the scoring functions in molecular docking. Even the activity predictions made through free energy perturbation (FEP) calculations show significant deviations when experimental data are lacking.

#### Complexity of biological systems

The complexity inherent in systems biology presents additional challenges for CADD. On the one hand, systems biology involves components at various levels and scales, including molecules, cells, tissues, and organs. CADD typically focuses on interactions and properties at the molecular level; however, it is crucial to consider how molecular information connects with higher-level organizations and physiological processes to more comprehensively interpret and predict drug effects and mechanisms of action^215^. On the other hand, biological systems are dynamic and involve processes such as metabolic pathways and cellular signaling. When designing drugs, the dynamic changes and regulatory mechanisms within the system need to be accounted for. However, there was no significant improvement in the accuracy of the prediction of NAFLD according to the ADMET data. This is primarily because the publicly available experimental data are extremely limited and insufficient to support the development of high-quality predictive models. Notably, single nucleotide polymorphisms (SNPs) can reveal individual susceptibilities to different diseases and sensitivities to drug metabolism at the genetic level, reducing confounding factors in complex biological systems and potentially facilitating more accurate target predictions^216–218^. Additionally, the recent rise of natural RCT experiments and Mendelian randomization studies used for causal inference in various systemic diseases also provides direction for identifying multiple targets for complex diseases^216,219^. Therefore, designing various preventive drugs for diseases with causal relationships is a promising prospective area of research.

#### Legal and ethical issues

CADD research involves human biological information and the design, development, and related clinical application of drugs. Therefore, it is crucial for researchers to adhere to the principles of research ethics and moral guidelines to ensure the rationality, safety, and reliability of human trials and clinical applications. This includes meeting the requirements for informed consent, privacy protection, fairness, and equity concerning human test subjects. Additionally, the application of CADD might involve controversial or risky areas, such as creating new toxic substances or therapeutic methods that have not yet been approved. Researchers bear ethical responsibility for their studies and applications and must therefore ensure proper regulatory and review mechanisms are in place.

### Novelty of the study

The main methods used in this study are bibliometric analysis and content analysis. Among other things, the bibliometric analysis method is an objective and empirical method that reveals the structure and trends of basic theories in the field of science and further identifies the groups of authors and research topics in this scientific field. Based on visualization and bibliometric methods, this study screens the core keywords for comprehensive and in-depth expansion, which demonstrates the history, current status and trends of the CADD research field from a macroscopic point of view and is innovative and scientifically valuable. Although this is a relatively new area of research, sufficient progress has been made in this area to further summarize the findings from the past to the present. However, since most reviews do not summarize the complete body of related research, there is still a need to synthesize the diversity of publications in this field in a systematic but accessible way. Therefore, this study uses bibliometrics and content analysis to review the current status, topical trends, and future developments in the field of CADD.

### Limitations of the study

Nevertheless, our research has several limitations. First, we included only articles with complete reports in the WOSCC database and did not use Google Scholar or Scopus, which might have led to the omission of some related literature. Second, concerning data completeness, only English-language literature was retrieved for this study, resulting in selection bias in the research. Additionally, some high-quality literature might not have been analyzed because it was published late or had a low number of citations.

## Conclusion

This review uses visualization and bibliometric methods to screen keywords and core authors for a comprehensive and in-depth expansion, innovatively demonstrating the current status and trends of CADD research in the field of medical research on a macro scale. These findings enable the academic and pharmaceutical communities to identify new directions and facilitate future application research on CADD in drug development. Importantly, computers are merely auxiliary tools that provide intuitive models for scientists to design drugs. The design process still requires the judgment and guidance of experienced scientists. Moreover, current CADD methods have many flaws and are in continuous development and refinement, requiring design and synthesis by organic chemists and validation through screening by pharmacologists. The present computational models do not yet reflect a comprehensive understanding of biological systems; breakthroughs have been achieved in only some specific areas. Thus, this creates obstacles to designing small-molecule drugs for any clinical need directly. However, as long as we continue moving in the right direction, it will be possible to realize drug design driven by CADD in the future.

## Ethical approval

This study does not include clinical patient information and does not involve clinical ethical content.

## Consent

This study does not include clinical patient information and does not involve clinical ethical content.

## Sources of funding

This research was financially supported by the National Natural Science Foundation of China (No. 82305159 and No. 82260914); Central Guided Local Science and Technology Development Funds Project (No. 2022ZDD03085); Jiangxi Technology Innovation Centre for Green Manufacturing of Traditional Chinese Medicine (No. 20222BCD43008).

## Author contribution

Z.W. and S.C.: literature search, data curation, formal analysis, and writing – original draft; Z.W.: investigation, methodology, and software; Y.W.: supervision and validation; H.X.: investigation, methodology, and software; F.L.: software; M.L.: validation and visualization; Y.Z. and Z.W.: funding acquisition, supervision, and writing – original draft; Y.G. and Z.W.: project administration, resources, and supervision.

## Conflicts of interest disclosure

The authors declare no conflicts of interest.

## Research registration unique identifying number (UIN)

This study does not include clinical patient information and does not involve clinical ethical content.

## Guarantor

Yue Gao and Zhenhui Wu are the guarantors for this article.

## Data availability statement

All data used in this review were obtained from Web of Science databases, which are represented in the articles, images, and tables. The authors do not have permission to share data.

## Provenance and peer review

This article has not been invited.

## Supplementary Material
