# Supplementary material for: Current perspectives and trend of computer-aided drug design: a review and bibliometric analysis
Source: Int J Surg. 2024 Mar 19;110(6):3848–78. doi: 10.1097/JS9.0000000000001289 (PMC11175770; doi:10.1097/JS9.0000000000001289)
Supplement: Supplementary file 1 [file js9-110-3848-s001.docx]

Dear editor,

Instructions for Authors on your journal's website, due to inability to access this website : [https://prisma-statement.org/PRISMAStatement/Checklist.aspx](https://prisma-statement.org/PRISMAStatement/Checklist.aspx)[.](https://edmgr.ovid.com/ijs/accounts/ifauth.htm.)

therefore, the files of PRISMA Flowchart and PRISMA Checklist cannot be generated.

best wishes

Zhenhui Wu
